# Probing the Design Rules for Optimizing Electron Spin Relaxation in Densely Packed Triplet Media for Quantum Applications

**DOI:** 10.1021/acsmaterialslett.4c01465

**Published:** 2024-12-19

**Authors:** Max Attwood, Yingxu Li, Irena Nevjestic, Phil Diggle, Alberto Collauto, Muskaan Betala, Andrew J. P. White, Mark Oxborrow

**Affiliations:** † Department of Materials and London Centre for Nanotechnology, Imperial College London, South Kensington Campus, Exhibition Road, SW7 2AZ London, United Kingdom; ‡ Department of Chemistry and Centre for Pulse EPR spectroscopy, Imperial College London, Molecular Sciences Research Hub, W12 0BZ London, United Kingdom

## Abstract

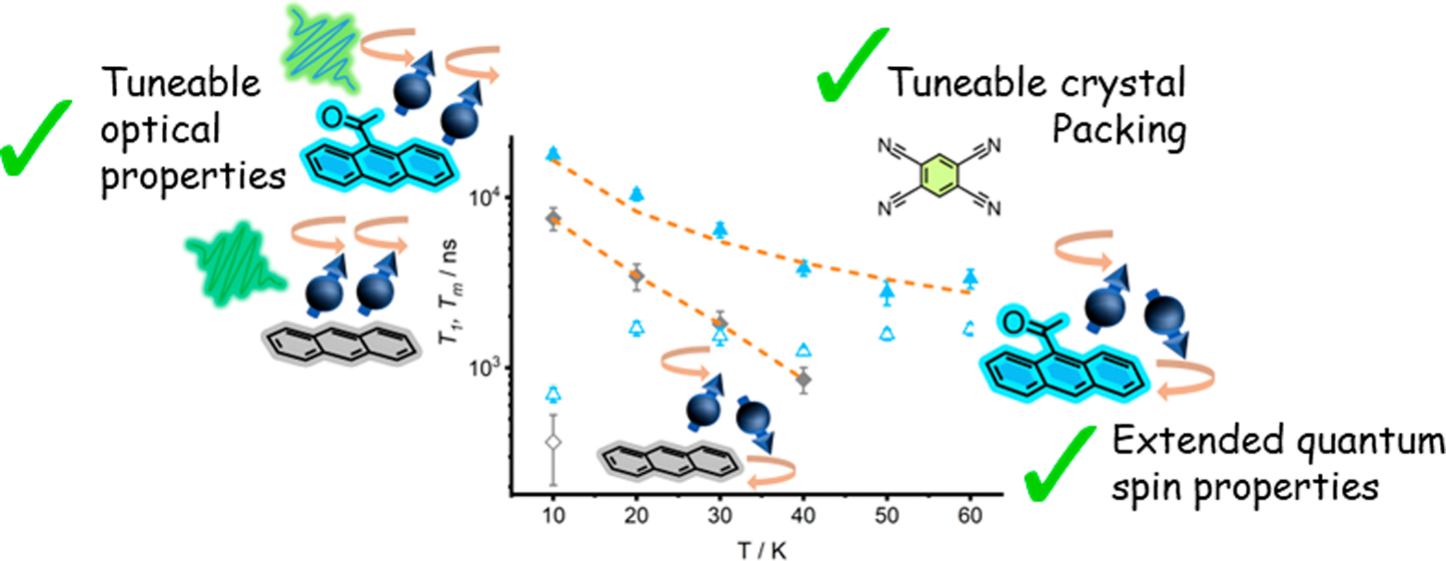

Quantum technologies using electron spins have the advantage of employing chemical qubit media with tunable properties. The principal objective of material engineers is to enhance photoexcited spin yields and quantum spin relaxation. In this study, we demonstrate a facile synthetic approach to control spin properties in charge-transfer cocrystals consisting of 1,2,4,5-tetracyanobenzene (TCNB) and acetylated anthracene. We find that the extent and position of acetylation control the degree of charge-transfer and the optical band gap by modifying crystal packing and electronic structure. We further reveal that while the spin polarization of the triplet state is slightly reduced compared to prototypical Anthracene:TCNB, the phase memory (*T*_m_) and, for 9-acetylanthracene:TCNB spin–lattice relaxation (*T*_1_) time, could be enhanced up to 2.4 times. Our findings are discussed in the context of quantum microwave amplifiers, known as masers, and show that acetylation could be a powerful tool for improving organic materials for quantum sensing applications.

Organic charge-transfer (CT) compounds are a diverse family of materials capable of exhibiting photoluminescence,^1−5^ photothermal conversion,^6^ thermal responsiveness^7^ and mechanoresponsiveness,^8,9^ conductivity,^10−12^ metastable electron spin,^13^ and magnetism.^14−16^ These materials are formed by self-assembly and crystallization following solvent evaporation,^17,18^ solvent diffusion,^19^ or sublimation methods.^20−24^ Their formation is governed by steric factors and electrostatic forces that occur between an electron-rich “donor” (D) and an electron-deficient “acceptor” (A). Therefore, the properties of CT cocrystals depend on the chemical constituents,^25−30^ and D–A stoichiometry.^31,32^ Among the myriad potential acceptors, 1,2,4,5-tetracyanobenzene (TCNB) has become a popular choice. This molecule can be matched with a range of acenes to form solvent-free cocrystals that are stable to air and light. Recently, due to their strongly spin-polarized triplet state following light excitation, CT cocrystals have been considered as candidates for quantum applications such as spin qutrits^13^ and quantum sensors known as “masers”.^33^

Masers are devices capable of amplifying microwave signals with extraordinary signal to noise.^34^ As such, masers have promising applications as sensor elements in radio- or microwave-based metrology applications,^35^ including EPR spectroscopy. Currently, NV-diamond, pentacene-doped *p*-terphenyl (Pc:PTP) and 6,13-diazapentacene-doped *p*-terphenyl (DAP:PTP) are the only materials found to yield either continuous or pulsed room-temperature maser signals without artificially boosting the gain.^36^ In the case of Pc:PTP, this is due to a combination of a high triplet quantum yield (ϕ_T_ ≈ 62.5%), strong triplet state spin polarization (P_X_:P_Y_:P_Z_ = 0.76:0.16:0.08) and robust quantum spin properties (*T*_1_ > 100 μs, *T*_m_ > 1 μs).^37,38^ However, the commercial prospects of these devices are limited by their dilute triplet spin densities and the need for strong pump light energies. For Pc:PTP, exceeding a 0.1% mol/mol concentration leads to pentacene aggregation and the propensity to form short-lived paramagnetic states associated with single fission.^39^ Improving maser operating conditions, therefore, requires simultaneous control over the crystal structure and the spin dynamics of the gain media, and mirrors the challenges of developing robust, optically addressable molecular media for quantum technologies.^40,41^

Much attention has been paid to the optimization of inorganic quantum systems wherein the spin properties are determined by the ligand and crystal field of inorganic spin centers,^42−46^ with limited attention on strategies for purely organic triplet spin materials.^36,47,48^ Previously Ng et al., attempted to use cocrystals of phenazine:TCNB as a maser gain medium.^33^ This approach had the advantage of high spin densities and straightforward growth of millimeter-sized crystals. However, phenazine:TCNB could not achieve autonomous maser oscillation due to rapid spin polarization decay. An effective method to chemically improve the ϕ_T_ and triplet lifetime is acetylation of an aromatic core. Acetyl groups have the advantages of stability and neutrality over other electron-withdrawing groups (e.g., −NO_2_, −COOH). In anthracene and pyrene, acetyl functionalization has enabled progressive improvements in triplet yield and adjustment of the optical band gap.^49,50^ Control over the optimal absorption frequency is vital, since longer wavelengths contain less energy per photon. Hence, more excited states can be generated per unit of pump energy, leading to reduced thermal loss, and thereby helping to ameliorate maser operation. However, such modifications on phenazine are challenging due to the presence of nucleophilic nitrogen groups. Therefore, we turned our attention to anthracene, a structurally similar molecule known to exhibit a spin-polarized triplet state in cocrystals with TCNB.^51^ Previous work by Philip et al. established that acetylanthracenes can exhibit ϕ_T_ values up to 100% in solution, improving upon the ∼70% exhibited by anthracene, with ϕ_T_ depending on spin–orbit coupling (SOC) through the stabilization of ^1^*n*π* states. However, the impact of acetylation on triplet sublevel spin polarization, *T*_1_, and *T*_m_ remains unknown.

In this study, we synthesize four CT cocrystals with acetylanthracenes and TCNB (Scheme 1) and characterize their structural, optical, and electron spin properties. 1-acetylanthracene (1-AAN) and 9-AAN were chosen, due to their high triplet yield, while 1,5- and 9,10-diacetylanthracene (1,5-DAAN and 9,10-DAAN, respectively) were chosen to look for further modulations in behavior. We determined that the position and degree of acetyl functionalization had profound effects on the optical band gap by modulating the donor strength of the anthracene cores. Furthermore, these materials demonstrated spin-polarization compatible with maser applications and marked improvements in *T*_1_ and/or *T*_m_, compared to the prototypical Anthracene:TCNB.

**Scheme 1:**
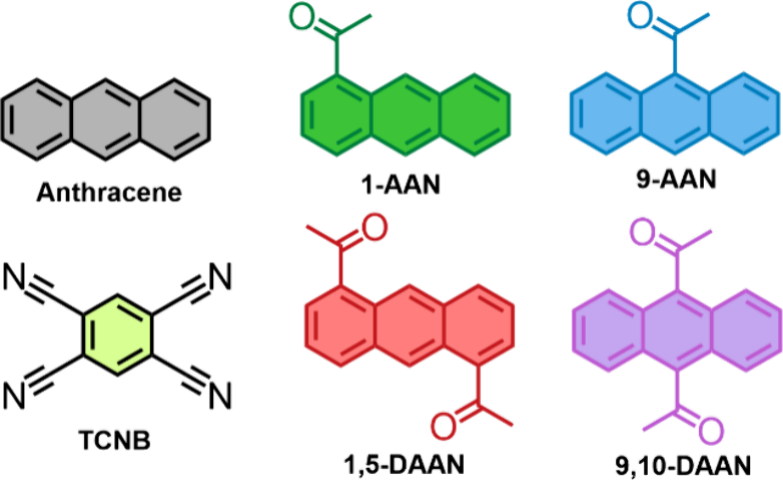
Structures of Molecules in This Report

The synthesis of each acetylanthracene derivative was straightforward. Although inevitably a mixture of products was obtained, control over mono vs disubstitution could be realized by conducting the experiment in an ice bath (see ESI). 9,10-DAAN precipitated as a gelatinous product, eventually solidifying after resting for a few days. To obtain optically dense materials with a well-ordered packing structure, we sought to incorporate these molecules into CT cocrystals with TCNB. Crystals were grown via codissolution in acetone followed by slow evaporation over 3 days. 1-AAN, 9-AAN, and 1,5-DAAN formed needle-shaped or rodlike crystals, respectively, while 9,10-DAAN did not coalesce into a crystalline form. As reported in the literature, 9-AAN:TCNB was found to be a polymorphic material.^52^ Our experiments predominately yielded orange needles with a minority of red blocks. Attempts to grow solely orange needles or red block crystals using mixtures of tetrahydrofuran and acetonitrile were unsuccessful. Therefore, we opted to remove the red block polymorph and focus on the majority orange material.

## Optical and Structural Characterization

**Figure 1:**
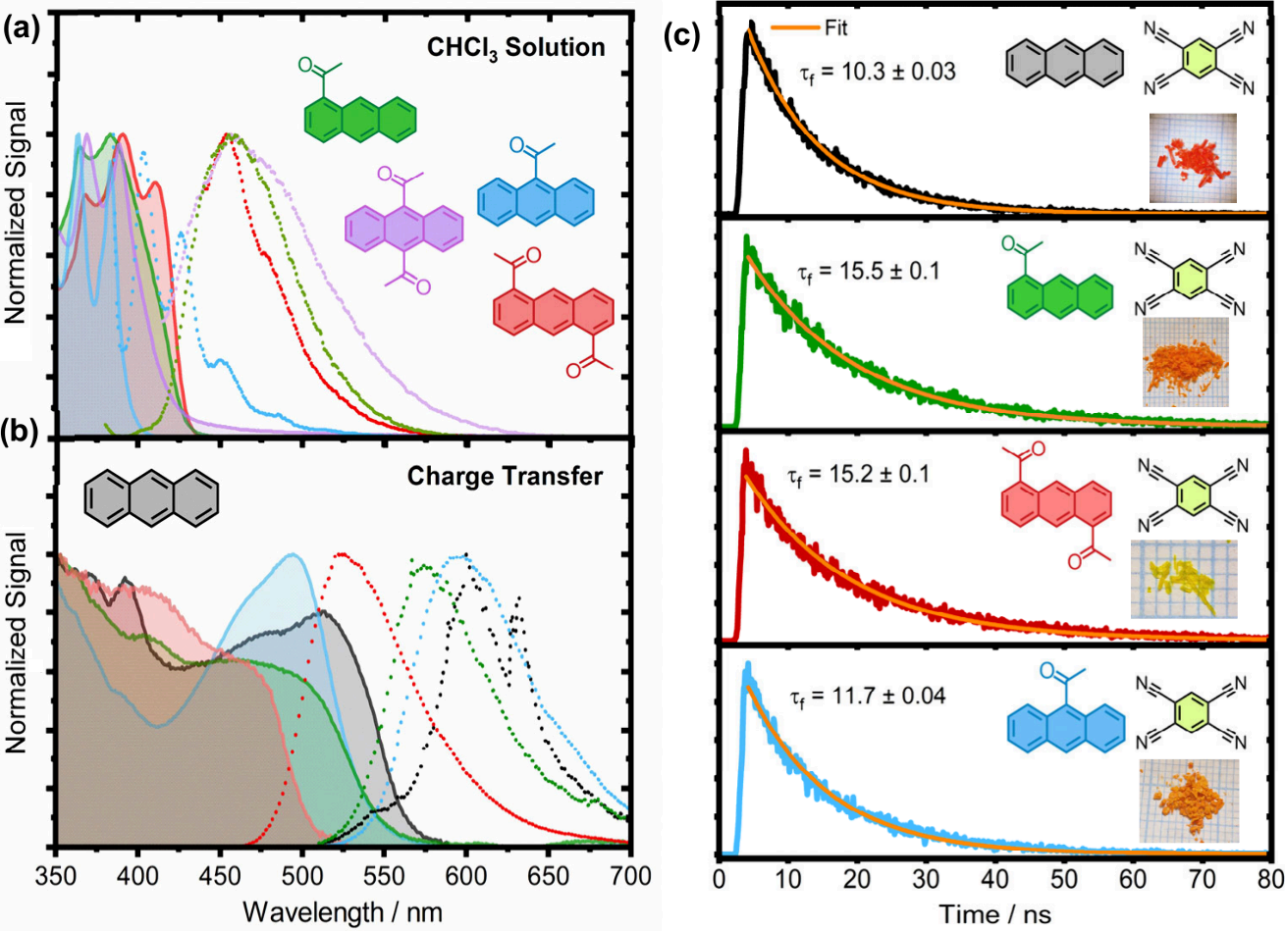
(a) UV/vis (solid lines) and fluorescence (dashed lines) spectra of four acetylanthracenes in CHCl_3_ solution (concentration = 10^–5^ M) and (b) drop-cast thin films of CT materials. (c) Time-correlated single photon counting traces for the four CT materials following excitation at 405 nm (inset shows images of crystallites).

Steady-state UV/vis and fluorescence spectroscopy were performed using each acetylanthracene in dichloromethane solution and using drop-cast films of the cocrystal. Solutions of the diacetylanthracene molecules exhibited a slight red shift in their absorption spectra, compared with solutions of monoacetylanthracene molecules, with all materials presenting a clear Frank–Codon structure (Figure 1a). All molecules have a room-temperature fluorescence response with shifted maxima corresponding to the absorption spectrum. Redshifted absorption is associated with stabilization of the LUMO due to either an extension of the chromophore π-system or reduced electron density due to the presence of peripheral electronegative functional groups.

Cocrystals all exhibited a significant redshift in their absorption and emission, reflecting the presence of low-lying LUMO levels introduced by weak CT interactions with the acceptor molecule, TCNB. All materials were found to absorb at higher frequencies compared to Anthracene:TCNB, suggesting that acetylation reduces the donor capacity of acetylanthracenes. 1-AAN:TCNB and 9-AAN:TCNB each show ∼120 nm redshifts in their absorption spectra while 1,5-DAAN:TCNB only exhibited a redshift of ∼50 nm, indicating that 1,5-DAAN is the weakest donor. This is consistent with the electron-withdrawing effect associated with π-conjugated acetyl groups (Figure S9). To probe their transient response, we also performed transient fluorescence spectroscopy across the spectra (see Figure 1c and Figure S1). Here, each cocrystal exhibited similar fluorescence decay times with the two materials expected to show the highest ϕ_T_, Anthracene:TCNB and 9-AAN:TCNB, being the fastest. We note that within the parameters of our experiments, we did not observe any signs of phosphorescence or delayed fluorescence via triplet–triplet annihilation (TTA) previously reported for Anthracene:TCNB.^53,54^ The nanosecond decays are consistent with intersystem (ISC) and are consistent with the reported work of Philip et al.^50^

**Figure 2:**
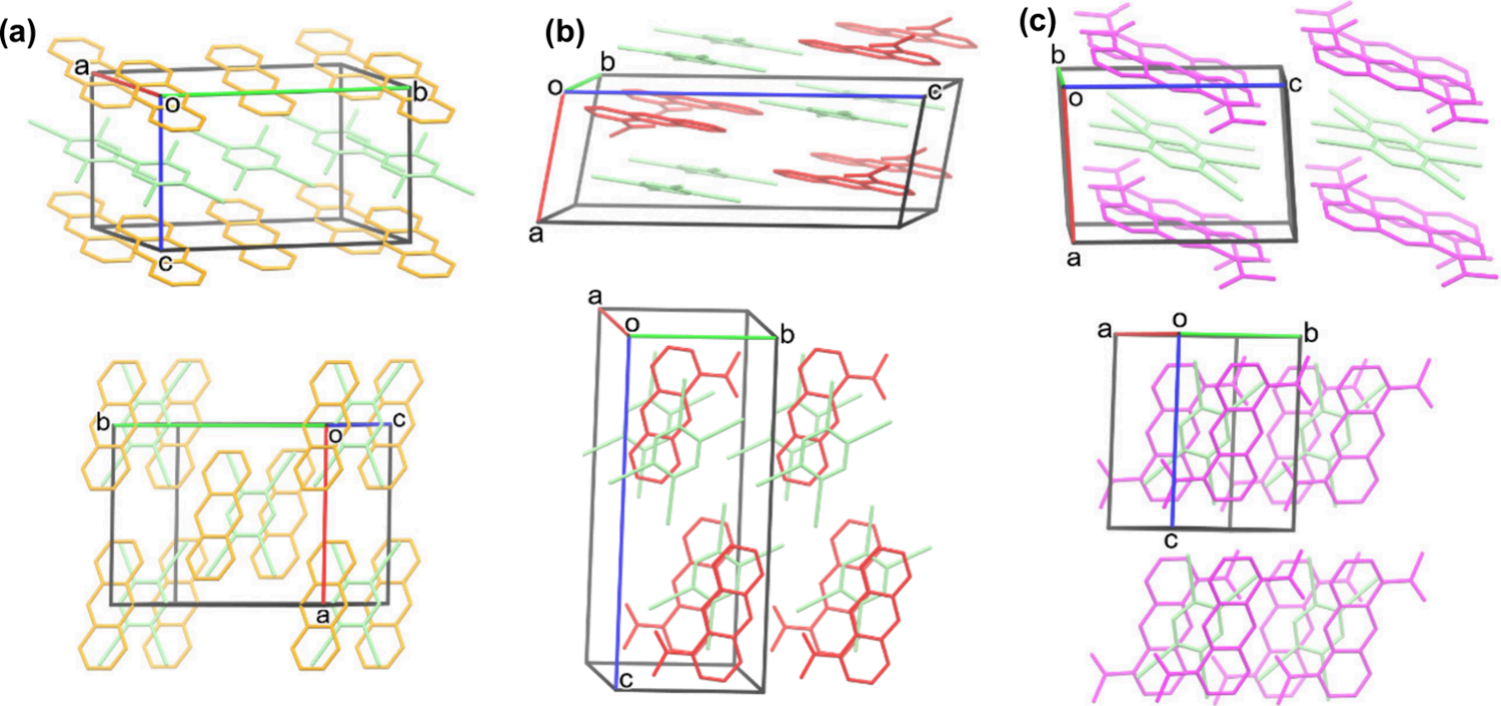
Packing of (a) Anthracene:TCNB; (b) 1-AAN:TCNB, and (c) 1,5-DAAN:TCNB. Molecules are presented in capped stick representation and captured using Mercury software (2022.1.0).

To understand the crystal packing of these materials, the structures of 1-AAN:TCNB and 1,5-DAAN:TCNB were solved by single-crystal X-ray diffraction. Crystals of 9-AAN:TCNB were found to exhibit significant twinning that prevented a definitive determination of their structure. 1-AAN:TCNB crystallized in the *P*1̅ space group and comprises a pseudomixed stack structure with columns of 1-AAN and TCNB molecules propagating along the *b*-axis, and π–π stacking along the *c*-axis (Figure 2b). Molecules of 1-AAN exhibit a 180° rotational disorder in an ∼78:22 ratio and interact through contacts between the acetyl-oxygen atoms and anthracene 9-position C–H groups.

While we were unable to solve the structure of 9-AAN:TCNB used in this study ourselves, the structures of both polymorphs have been reported Wang et al.^55^ In each, 9-AAN:TCNB presents with a mixed packing motif, however, 9-AAN molecules exhibit several common contacts between acetyl-CH_3_ groups and anthracene and TCNB rings. Hence, a reasonable explanation for our inability to isolate cocrystals with 9,10-DAAN is that the acetyl groups sterically prevent π–π CT interactions. Furthermore, homomolecular contacts imply that neighboring triplet moieties have the spatial and electronic means to interact. Such interactions can lead to reduced spin polarization lifetimes via competing magnetic dipole interactions or triplet–triplet annihilation (TTA).^56^ However, TTA also requires the appropriate electronic structure such that two *T*_1_ states can fuse to generate an S_1_ adiabatically. Importantly, the acetyl groups of 1-AAN and 1,5-DAAN are nearly planar with the anthracene rings, but nearly perpendicular in 9-AAN. Therefore, 9-AAN:TCNB should exhibit stabilized *n*π*-states identified previously for 9-AAN that are believed responsible for the higher ϕ_T_.^50^

## Density Functional Theory

To further understand the photophysical properties of these molecules and their cocrystals, we conducted TD-DFT calculations to estimate the energies of the excited singlet and triplet states. Typically, a high ϕ_T_ is realized by strong SOC and energetic proximity between singlet and triplet states. For acetylanthracene molecules, the singlet and triplet energies were estimated by performing a singlet or triplet geometry optimization in the gas phase, followed by TD-DFT calculations to determine optical excitation energies. This approach was able to closely reproduce the optical band gap trend observed in absorption data whereby diacetylanthracenes have a reduced S_0_ → S_1_ energy gap compared to monoacetylanthracenes, but with an absolute difference in calculated and experimental values of ∼5%–10% (see Figure S9a and Table S3). The same holds for the *T*_1_ states, which are slightly closer to S_1_ for diacetylanthracenes compared to monoacetylanthracenes. Our geometry optimizations also confirmed that the acetyl groups of 1-AAN and 1,5-DAAN are nearly planar with the anthracene rings, whereas the acetyl groups of 9-AAN and 9,10-DAAN are rotated ∼90° out-of-plane. As a result, acetyl orbital contributions are less prominent on the HOMO and LUMO for 9-AAN and 9,10-DAAN, with the LUMO state (associated with S_1_) having *n*π* character (Figure S9b). These results are also consistent with geometries displayed in the crystal structures reported for each polymorph of 1-AAN (and see Figure S8),^57^ 9-AAN,^50,58^ 1,5-DAAN,^57,59^ 9,10-DAAN,^60^ and various other constitutional isomers.^60^ Previously, the faster ISC rate of 9-AAN, compared to 1-AAN has been attributed to the stabilization of singlet *n*π* states compared to ππ* states, leading to strong SOC.^50^ Indeed, TD-DFT calculations at the B3LYP/def2-SVP level indicate that 1,5-DAAN and 9,10-DAAN maintain a comparable SOC between singlet and triplet states to 1-AAN and 9-AAN (see Tables S4–S8), respectively, despite each having additional acetyl groups.

**Figure 3:**
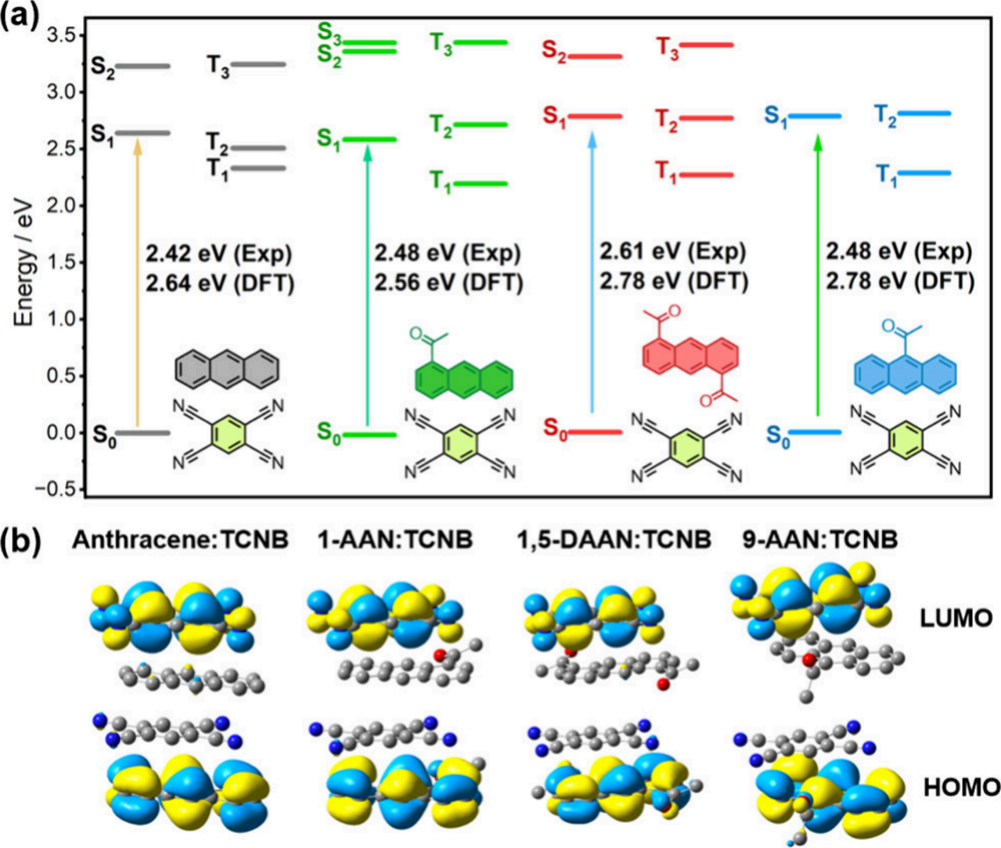
(a) Singlet and triplet state energies of cocrystal dimers with singlet optical band gaps determined using UV-vis spectroscopy (Exp) and TD-DFT, and (b) their highest occupied molecular orbitals (HOMOs) and lowest unoccupied molecular orbitals (LUMOs).

TD-DFT calculations were also performed on cocrystal dimers using their single geometries as the ground state at the CAM-B3LYP 6-311+G(d) level^61^ (see the Electronic Supporting Information (ESI) for more details). These calculations were broadly effective at reproducing the trends observed by UV/vis spectroscopy with the S_0_ → S_1_ optical excitation energy of all materials within 3%–12% of their experimental values (Table S3), most consistent with the performance expected for TD-DFT.^62^ However, while the S_0_ → S_1_ excitation energy of 1,5-DAAN:TCNB was larger than 1-AAN:TCNB, as expected, the latter was predicted to have a smaller band gap than Anthracene:TCNB and 9-AAN:TCNB. This likely reflects the inability of DFT approaches to accurately estimate the exchange-correlation energy for systems with a stronger CT contribution. Nevertheless, these calculations clearly reveal that the *T*_1_ state in CT dimers is significantly destabilized compared to pure acetylanthracenes (Figure 3a). As a result, the S_1_ state is energetically closer to both *T*_2_ and *T*_1_, with relatively small singlet–triplet energy differences (Δ*E*_S_1_–T*_n_*_, Table S3). However, unlike the neat materials, these calculations suggest that both S_1_ and *T*_2_ are located on the TCNB moiety and exhibit largely ππ*-orbital character (Figure S10). Hence, S_0_ → S_1_ transitions correspond to an intermolecular CT event and hence the triplet states useful for masing are likely formed by ISC into the *T*_2_ state, followed by relaxation to *T*_1_. Alternatively, triplets could be generated by S_1_ → *T*_1_ relaxation; however, the relatively large predicted Δ*E*_S_1_–T_1__ renders this pathway less energetically favorable. It is also possible that the population of a CT state would lead to mobile excitons incapable of masing, and their recombination would statistically repopulate singlet and triplet states equally and contribute to a reduced spin polarization magnitude. The contributions of delocalized and localized triplet states should be detectable using EPR spectroscopy.

## EPR Spectroscopy

To understand how the acetylation of anthracene and its incorporation in CT cocrystals affects the spin properties of the triplet states, we performed X-band time-resolved electron paramagnetic resonance (trEPR) spectroscopy under photoexcitation. To measure triplet signals of the neat acetylanthracenes, each was doped under anaerobic conditions into *o*-terphenyl (concentration = 0.1% mol/mol), which forms an optically transparent viscous state upon melting that is metastable at room temperature.^63,64^ However, we were unable to record any signals despite their expected high ϕ_T_.^50^ One explanation could be that their polarization lifetime is too fast for our instrument’s 200 ns response time. We note that the triplet lifetime of 1-AAN and 9-AAN in chloroform has been measured at just 6.9 and 4.5 μs.^50^

**Figure 4:**
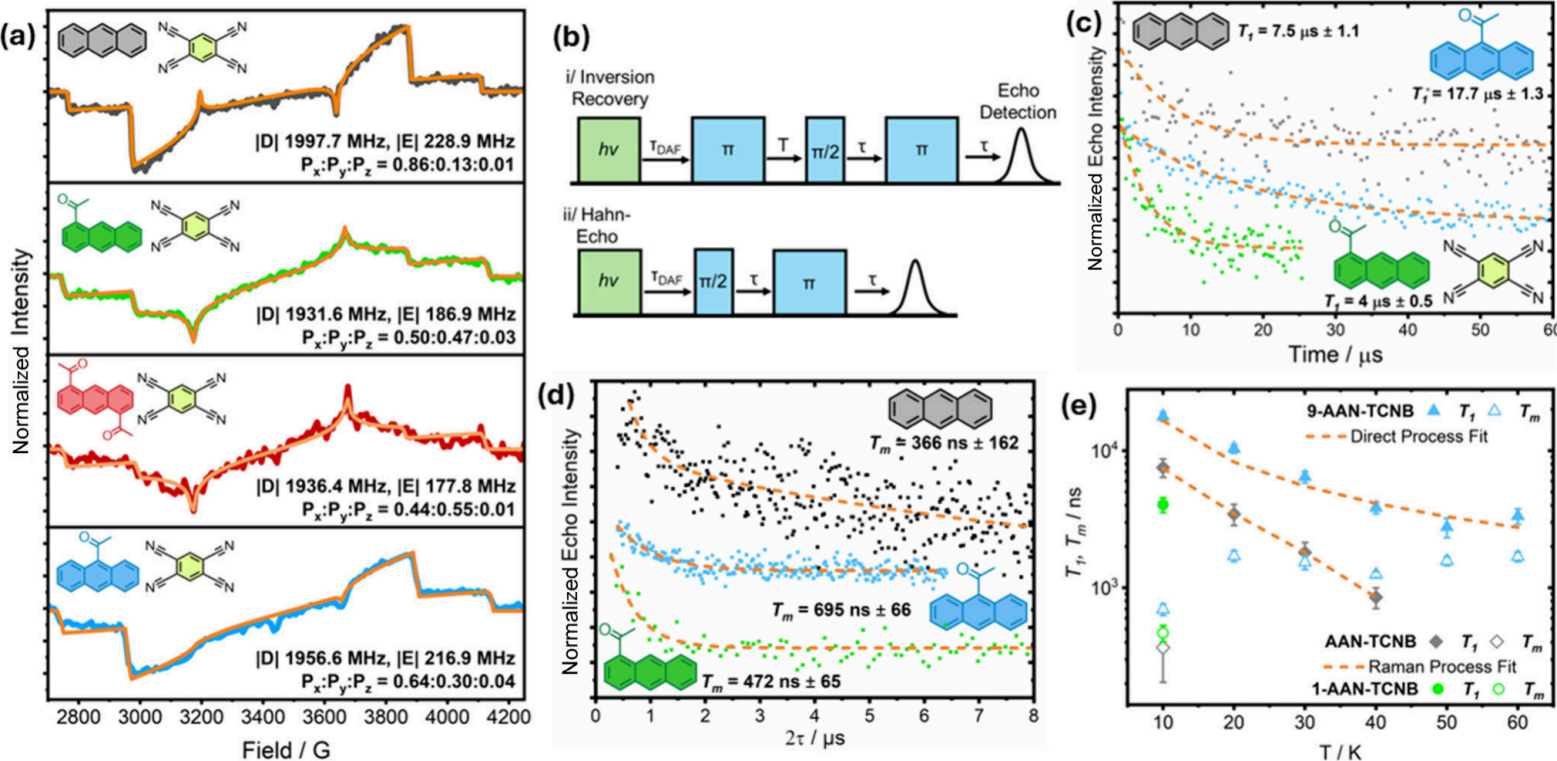
(a) Room-temperature trEPR spectra for powders of Anthracene:TCNB, 1-AAN:TCNB, 1,5-DAAN:TCNB, and 9-AAN:TCNB taken 400 ns after laser flash. (b) Spin inversion and Hahn-Echo pulse sequences with the delay after flash (τ_DAF_) equal to 0. (c) Measurements of *T*_1_, and (d) *T*_m_ at 10 K fitted with monoexponential decay functions (orange dashed lines). (e) Variable temperature measurements with *T*_1_, fitted according to direct and Raman relaxation processes. Pulsed experiments were performed at 2950 G for 9-AAN:TCNB and 1-AAN-TCNB, and 3900 G for Anthracene:TCNB to approximate the canonical *T*_*x*_–*T*_*y*_ transition. All data was collected using 500 nm light, 3.5–4.5 mJ/pulse, 5–7 ns pulses.

The cocrystal samples were ground into powders and excited at the leading-edge absorption measured by UV/vis spectroscopy. These measurements returned spin-polarized triplet signals for each cocrystal consistent with ISC (Figure 4a). Anthracene:TCNB and 9-AAN:TCNB exhibited the most intense signals, which is consistent with their expected high ϕ_T_ and faster fluorescence decay. It is also worth noting that these materials also presented a small center field signal in their respective trEPR traces again consistent with stronger CT. Whether this CT interaction encourages ISC remains uncertain, however, Anthracene:TCNB is known to generate mobile triplet excitons following photoexcitation.^65,66^ For quantum applications, it is important to generate a stable unpaired spin state. Hence, our approach of using acetylation to modify the CT interaction for these densely packed triplet media could be an important tool for future investigations.

The zero-field splitting (ZFS) parameters were similar between the acetylanthracenes which presented with |*D*| and |*E*| values slightly reduced, compared to Anthracene:TCNB, likely reflecting the larger delocalization across the acetyl groups. Interestingly, the triplet sublevel populations for 1-AAN:TCNB, and 1,5-DAAN:TCNB showed similar populations in their respective *T*_*x*_ and *T*_*y*_ states, with minimum *T*_*z*_ occupation for each material. Hence, the ideal ZF maser frequency for Anthracene:TCNB is |*D*| + |*E*|, ∼2227 MHz. For acetylanthracenes, however, similar spin polarization in *T*_*x*_ and *T*_*y*_ suggests that either *T*_*x*_ → *T*_*z*_ or *T*_*y*_ → *T*_*z*_ transitions might be suitable for masing. To reach the so-called maser threshold, a system must exhibit sufficient cooperativity to overcome losses from the microwave cavity. As reported by Breeze et al.,^67^ for steady-state conditions, the pump power required to sustain continuous maser oscillation can be expressed as
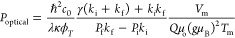
1where *P*_optical_ is the minimum pump energy needed to sustain maser oscillation, λ is the pump wavelength, *k* is the optical coupling efficiency, ϕ_T_ is the intersystem crossing yield, *P*_f_ and *P*_i_ are the populations in the final and initial triplet sublevels involved in the maser transition, μ_o_ is the permittivity of free space, μ_B_ is the Bohr magneton, γ is the spin–lattice relaxation rate between the relevant sublevels, *k*_f_ and *k*_i_ is the triplet sublevel depopulation rate, *V*_m_ is the magnetic mode volume, and *Q* is the cavity quality factor.

Equation 1 shows that a deficit in spin polarization can be compensated by a higher ϕ_T_, or longer spin–lattice relaxation times and *T*_m_. Therefore, to further evaluate their spin dynamics, we performed inversion recovery and Hahn-echo pulsed EPR experiments to determine *T*_1_ and *T*_m_, respectively (Figure 4b). To optimize signal-to-noise and lower spin lifetime errors, measurements were initially performed at magnetic fields corresponding to the canonical Y-positions (∼295 mT, Figure S7) and 10 K. Unfortunately, 1,5-DAAN:TCNB remained too weak to reliably measure even under these conditions (Figure S7). This is likely due to a reduced triplet yield and spin-polarization compared to the other materials. Echo decay traces were fitted using a single exponential and revealed that acetylated materials exhibited longer *T*_m_ values than Anthracene-TCNB, with 9-AAN:TCNB also demonstrating an ∼2.4-fold improvement in *T*_1_ (see Figures 4c and 4d). Under these conditions, and assuming similar *k*_f_ and *k*_i_, then according to eq 1, the reduction in spin polarization density would only be significantly compensated, particularly for 9-AAN:TCNB. To further understand the source of these differences in *T*_1_ and *T*_m_, measurements were performed up to 60 K for 9-AAN:TCNB. Low signal-to-noise prevented echo measurements above 10 K for 1-AAN:TCNB and Anthracene:TCNB, except for *T*_1_, which we managed to acquire up to 40 K for the latter. The temperature dependence of *T*_1_ exhibited by 9-AAN:TCNB could be fitted only considering a direct relaxation process, which is usually associated with spin relaxation below 10 K. This could suggest a higher Debye temperature and/or a correspondingly sparse vibrational state density, which limits two-phonon Raman/Orbach-type relaxation. The increased temperature dependence exhibited by Anthracene:TCNB above 20 K required fitting according to either the Orbach relaxation, with an excited state energy constant (Δ) of 182 ± 12, or Raman relaxation, with a characteristic power of *T*^5^. *T*_1_ is ultimately determined by the distribution of phonon energies and the apparent presence of two-phonon relaxation in Anthracene:TCNB indicates the presence of low-lying virtual or excited electronic states. TD-DFT calculations predicted a *T*_2_ state within 0.18 eV (∼2000 K) of *T*_1_ for Anthracene:TCNB, corresponding to a vibrational frequency, and 0.92 eV for 9-AAN:TCNB. Orbach-type fitting of *T*_1_ temperature dependence suggests that the energy of the excited state is 182 ± 12 K, which does not align with the DFT calculations and otherwise makes Orbach relaxation an unreasonable candidate for these materials. Instead, we find that the most likely mechanism is Raman relaxation involving a lower energy virtual state, characterized by its 1/*T*^5^ dependence. Its seeming absence in 9-AAN:TCNB within the measured temperature range suggests the intermolecular interactions and differences in crystal packing could change the phonon energy distribution within the lattice. We also note that unusually, 9-AAN:TCNB exhibited a clear and increasing *T*_m_ up to 60 K, running contrary to *T*_1_. Poor signal-to-noise for these materials prevented a more comprehensive temperature-dependent exploration; however, similar behavior has been attributed in the literature to environmental factors dominated by translational diffusion of solvent protons.^68^ Since our system is solid, this explanation is not sufficient. One possible explanation could be triplet diffusion. *T*_m_ is partially dependent on electron dipole coupling and spin flipping,^13,69^ and the high concentration of localized triplet states formed immediately after the laser pulse will be a significant source of decoherence. In these CT materials, increasing the temperature could enable a higher portion of the initial excited states to become delocalized excitons, thereby effectively reducing the number of localized triplet states in a similar manner to increasing the delay after the laser pulse. One problem with this theory is the implicit assumption that the system is within the exciton hopping threshold, which is associated with reducing the spin decoherence.^13^ This will form the basis of further investigations. Since the *T*_m_ values of 1-AAN:TCNB and 9-AAN:TCNB are longer than those of Anthracene:TCNB, it is reasonable that changes in the crystal packing resulting from acetyl groups positively modify the local spin bath. Indeed, the distances of closest approach between anthracenyl moieties in the structures of each material are ∼2.5, ∼2.69, and ∼2.4 Å, respectively, correlating with the improvement in *T*_m_. Future investigations could utilize deuterated spin materials to distinguish the impact of intramolecular and intermolecular nuclei.

In summary, a series of optically dense and chemically tunable CT cocrystals have been synthesized and investigated for their ability to generate triplet states relevant to quantum applications. By employing a simple and versatile chemical approach of acetylation, we have shown it is possible to logically tune the optical band gap, triplet yield, the electronic and crystal packing structures, and as a result, the degree of triplet state spin-polarization and quantum spin dynamics. This study adds an important chemical tool to optimize quantum spin parameters,^42^ and it paves the way for further exploration of additional chemical strategies to realize robust organic spin-based materials for quantum technologies. While the prospect of autonomous masing using acetylanthracene cocrystals and the native quality factor of, for example, a strontium titanate dielectric resonator (*Q* ≈ 2000) is perhaps unrealistic, the application of acetylation to enhance the properties of, for example, tetracene or pentacene-based materials is an enticing idea, as is the exploration of other acyl/electron-withdrawing functional groups. For example, through acetylation, traditionally poorly soluble linear acenes should become more soluble in common organic solvents and more stable through steric hindrance of reactivity at the 6,13- or 5,12-positions of pentacene and tetracene, respectively. While their polarity and shape would likely prohibit crystal doping in a typical *p*-terphenyl host using a Bridgmann growth approach, these materials could be incorporated into a universal host, such as 1,3,5-tri(naphthyl)benzene cocrystals,^70^ with, for example, TCNB or 7,7,8,8-tetracyanoquinomethane (TCNQ). For example, cocrystals of tetracene:TCNQ exhibit strong CT interactions, which lead to a nonradiative internal conversion between S_0_ and S_1_, due to the energy gap law and significantly reduced fluorescence and triplet quantum yields.^71^ However, here acetylation has proven to be an effective tool for modulating the CT interaction and the optical band gap. Such band gap modification could also be useful to modify materials with long *T*_1_, but which may require damaging UV-light to address, such as picene.^72^ Therefore, by modulating the crystal structure, optical band gap, ϕ_T_, triplet sublevel populations, *T*_1_ and *T*_m_ in one versatile approach of acetylation, it should be possible to minimize the pump energy required for maser oscillation and improve the performance of alternative organic molecule-based quantum technologies.

## Supporting Information Available

Further data including fluorescence spectroscopy, DFT details, spin–orbit coupling calculations, and EPR spectroscopy (PDF)
