# Supplementary material for: Probing the Design Rules for Optimizing Electron Spin Relaxation in Densely Packed Triplet Media for Quantum Applications
Source: ACS Mater Lett. 2024 Dec 19;7(1):286–94. doi: 10.1021/acsmaterialslett.4c01465 (PMC11707738; doi:10.1021/acsmaterialslett.4c01465)
Supplement: Supplementary file 1 — tz4c01465_si_001.pdf [file tz4c01465_si_001.pdf]

Supplementary Information

Probing the design rules for optimizing electron spin relaxation in densely packed triplet media for quantum applications

Max Attwood[1*], Yingxu Li[1], Irena Nevjestic[1], Phil Diggle[1], Alberto Collauto[2], Muskaan Betala[1], Andrew J. P. White[2], Mark Oxborrow[1]

1/ Department of Materials and London Centre for Nanotechnology, Imperial College London, South Kensington Campus, Exhibition Road, SW7 2AZ, London, UK

2/ Department of Chemistry and Centre for Pulse EPR spectroscopy, Imperial College London, Molecular Sciences Research Hub, W12 0BZ, London, UK

Contents

Experimental Details 2

Transient Fluorescence Spectroscopy 3

Synthesis Details 5

EasySpin Fitting Details and Script 9

Pulsed EPR Data and Fitting 10

Single Crystal X-ray Diffraction 11

Additional DFT Calculations 13

Spin-orbit Coupling Calculations 15

References 19

Experimental Details

UV/Vis spectroscopy of the acetylanthracenes was performed using a sealed 1 cm quartz cuvette filled with samples diluted in chloroform to 10^{-5} M. Co-crystals were too optically dense to measure directly, hence spectra were measured using drop cast films on 1 cm^2 glass substrates. The feedstock solutions were diluted to 10^{-4} M and 100 µl was deposited on a glass and allowed to evaporate in air. Data was measured using an Agilent Cary 5000 UV/Vis/NIR spectrophotometer. Fluorescence emission spectroscopy was performed using a Horiba Jobin-Yvon Spex Fluorolog-3 fluorimeter, with emission and excitation slit widths set to 2 nm.

Diffraction data for crystals of **Anthracene:TCNB**, **1-AAN**, **1-AAN:TCNB** and **1,5-DAAN:TCNB** were collected using Agilent Xcalibur 3 E (**Anthracene:TCNB** and **1,5-DAAN:TCNB**) and Agilent Xcalibur PX Ultra A (**1-AAN** and **1-AAN:TCNB**) diffractometers using Mo-Kα and Cu-Kα radiation respectively, and the structures were solved and refined using the OLEX2,[1] SHELXTL47 and SHELX-2013[2,3] program systems. CCDC 2313864 to 2313866 and 2347419.

Time-resolved EPR spectroscopy was performed using a Bruker ELEXSYS-II E500T spectrometer (X-band) equipped with Bruker ER4118X-MD-5W resonator and coupled with 20 Hz Q-switched Nd:YAG laser (Spectra-Physics INDI-HG-20S) pumped broadband OPO (GWU, primoScan/ULD 120). Measurements were performed at room temperature at 450 nm (for **1,5-DAAN:TCNB**) or 500 nm (for **Anthracene:TCNB**, **1-AAN:TCNB** and **9-AAN:TCNB**) with ~5mJ laser pulse energy (pulse length 5-7 ns). Crystals were initially ground into fine powders and decanted into a 4 mm O.D. Wilmad quartz (CFQ) EPR tube. The attenuation was set to 0.05 mW and required 1500 scans. The data was then exported and analyzed using the MATLAB EasySpin (V6.0.0 dev.51) work package (see ESI for details).[4]

Pulse EPR experiments were performed on a Bruker Elexsys E580 spectrometer equipped with an ER 4118X-MD5 dielectric ring resonator (Bruker) and a 1 kW traveling-wave tube amplifier (Applied Systems Engineering Inc., Model 117X); a closed-circuit Helium cryostat with front optical access (Cryogenic Ltd.) was used to control the temperature of the resonator and of the sample. To enhance the sensitivity, the microwave resonator was installed on a cryogenic HEMT probehead (Amplify My Probe Ltd.) containing a 36 dB low-noise preamplifier (Low Noise Factory AB, model LNF-LNC4_16C) and a 6 dB directional coupler (Pasternack Enterprises, Inc.).[5] The light pulses were generated using an Aurora II Integra laser (Litron Lasers Ltd., model A23-39-21) containing a Nd:YAG nanosecond pump source and a Type II BBO broadband OPO; the pulse length for this system is 5 – 7 ns with a repetition rate of 21 Hz. The emission wavelength was tuned to 500 nm and the output energy was reduced to 3.5 – 4.5 mJ per pulse using an absorptive ND filter (Thorlabs Inc.). The sample was irradiated through the optical window of the cryostat (Spectrosil B fused silica). The pulse generation and EPR signal acquisition was triggered from the laser power supply unit. The trigger signal was delayed (delay after flash, DAF) in order to be at the maximum of the photo-induced transient signal; this calibration was performed using a sample of pentacene in 1,3,5-tri(1-naphtyl)benzene.51 Echo-detected field-swept EPR (EDFS-EPR) spectra were recorded using the pulse sequence $\pi/2 - \tau - \pi - \tau - echo$ with a fixed inter-pulse delay τ = 200 ns; a 2-step phase cycle was applied to the $\pi/2$ pulse. The same pulse sequence was used to record Hahn echo decay traces upon increasing the inter-pulse delay τ from 200 ns; a 2-step phase cycle was applied to the $\pi/2$ pulse. Inversion recovery experiments were performed using the pulse sequence $\pi - T - \pi/2 - \tau - \pi - \tau - echo$ with a 2-step phase cycle on the $\pi/2$ pulse; τ was set to 200 ns and T was incremented from 330 ns. For all the experiments the lengths of the $\pi/2$ and π pulses were set to 16 ns and 32, respectively, and the 2-pulse echo signal was integrated over a 100 ns window centered at the echo maximum. For each sample, the microwave power was optimised for maximum echo signal. To correct for the resonator background, for all the experiments traces with and without photoirradiation were recorded; the latter ones were then subtracted from the former ones.

Transient Fluorescence Spectroscopy

Transient fluorescence spectroscopy was obtained using a home-built setup as shown in **Fig. S1** below. The supercontinuum laser (NKT Photonics SuperK FIANIUM) was set to a repetition rate between 5 – 10 MHz. The light is directed to a 5x reflective beam expander and then translated to a custom monochromator for spectral selection. Spectral selection ensures that any wavelength between 400 and 700 nm has a bandwidth (FWHM) < 4 nm.

After spectral selection, the wavelength of light is directed towards a dichroic such that light is reflected towards a 10x microscope objective. The dichroic is a longpass filter such that the laser wavelength is reflected only in order to reject the excitation wavelength during spectroscopy.

The fluorescence is obtained by reflection and is therefore captured by the objective and directed towards the spectrometer (Andor Kymera 328i). The entrance slit to the spectrometer was set to 25 um, and spectroscopy was obtained using a 300 l/mm grating.

For data acquisition, the hybrid PMT (Becker & Hickl HPM-100-07) is connected to a streaming time-to-digital converter (Swabian Instruments TimeTagger Ultra). Data was collected with a dwell time such that sufficient signal was collected for data fitting routines (see Table S1). The instrument response function (IRF) was measured to be < 0.1 ns. The decay histogram data was collected into 0.1 ns temporal bins.

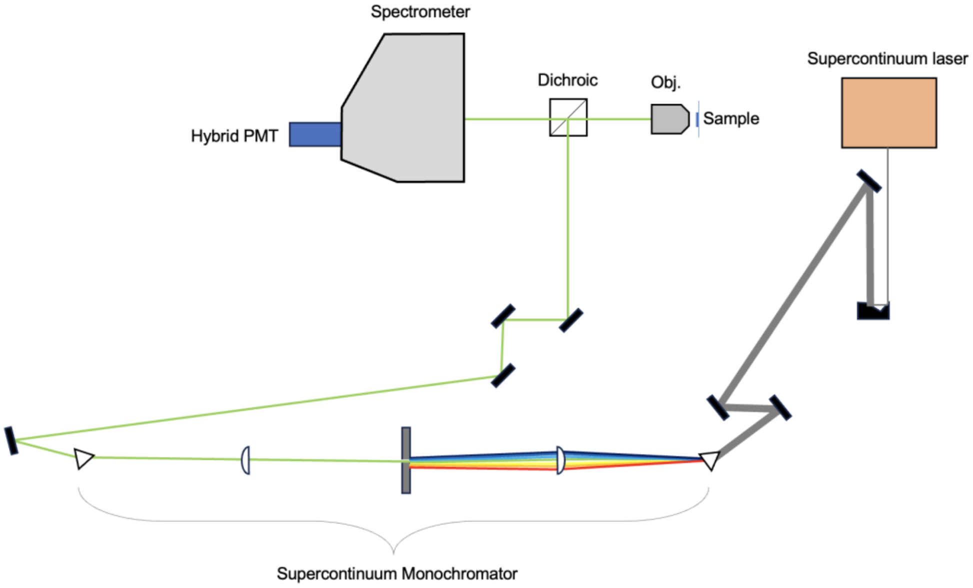

Figure S1: Schematic of the homebuilt hyperspectral microscope for transient fluorescence spectroscopy.

Table S1: Acquisition parameters for each sample.

Sample	Excitation Wavelength / nm	Laser Rep. Rate / MHz	Detection window / nm	Dichroic selection	Dwell time / s
**1-AAN:TCNB**	493	5.6	540 - 840	540 nm	15
**1,5-AAN:TCNB**	466	5.6	475 - 745	470 nm	20
**9-AAN:TCNB**	492	5.6	540 - 840	540 nm	10

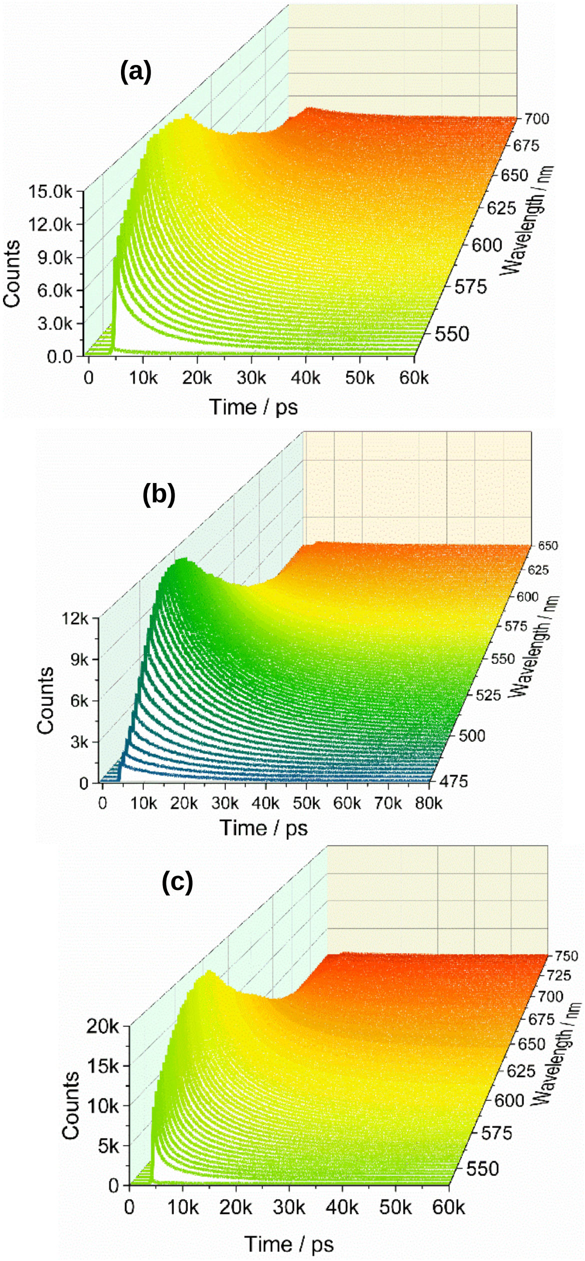

Figure S2: Transient fluorescence spectra for (**a**) **1-AAN:TCNB**, (**b**) **1,5-DAAN:TCNB**, and (**c**) **9-AAN:TCNB**.

Synthesis Details

Synthesis of acetylanthracenes was performed using modified procedures from the literature.[7,8]

1-AAN and **1,5-DAAN** were synthesized at room temperature in chloroform using an aluminium chloride ($AlCl_3$) Lewis acid and acetyl chloride. The products were extracted with ethyl acetate, washed with 5% Na_2CO_3, and separated by column chromatography using 1:1 dichloromethane to petroleum ether as eluent. Their NMR in $CDCl_3$ spectra matched those in the literature.

9-AAN and **9,10-DAAN** were synthesised using an α-Fe_2O_3 catalyst which had been activated by heating to 200°C for 3 days. A solution of anthracene and acetyl chloride was then added. The solution was stirred for an hour and then hydrolyzed using HCl or H_2SO_4 solutions. The products were extracted with ethyl acetate, washed with 5% Na_2CO_3, and separated by column chromatography using separated over silica gel using 5:1 ethyl acetate to petroleum ether.

9-acetylanthracene (**9-AAN**) assignments. ^{1}H NMR (400 MHz, CHLOROFORM-*D*) δ 8.48 (s, 1H), 8.03 (dd, *J* = 8.3, 1.7 Hz, 2H), 7.92 – 7.73 (dd, 2H), 7.65 – 7.39 (m, 2H), 2.81 (s, 3H). ^{13}C NMR (400 MHz, CHLOROFORM-*D*) δ 208.20, 136.83, 131.18, 128.91, 128.29, 126.86, 126.70, 125.58, 124.42, 33.94. ^{13}C NMR (400 MHz, CHLOROFORM-*D*) δ 128.91, 128.29, 126.86, 125.58, 124.42, 33.93.

9,10-diacetylanthracene (**9,10-DAAN**) assignments. ^{1}H NMR (400 MHz, CHLOROFORM-*D*) δ 7.85 (dd, *J* = 6.7, 3.2 Hz, 1H), 7.64 – 7.47 (m, 1H), 2.81 (s, 2H). ^{13}C NMR (400 MHz, CHLOROFORM-*D*) δ 207.63, 138.39, 127.66, 126.94, 126.09, 125.01, 33.97. ^{13}C NMR (400 MHz, CHLOROFORM-*D*) δ 126.93, 125.01, 33.96.

1-acetylanthracene (**1-AAN**) assignments. ^{1}H NMR (400 MHz, CHLOROFORM-*D*) δ 9.56 – 9.32 (m, 1H), 8.45 (s, 1H), 8.17 (dd, *J* = 8.5, 0.9 Hz, 1H), 8.11 – 8.03 (m, 1H), 8.00 (dd, *J* = 7.0, 1.1 Hz, 1H), 7.55 – 7.43 (m, 3H), 2.81 (s, 3H). ^{13}C NMR (400 MHz, CHLOROFORM-*D*) δ 201.64, 134.03, 133.13, 132.05, 131.63, 129.74, 129.30, 127.85, 127.73, 127.01, 126.27, 126.10, 125.89, 123.54, 29.91. ^{13}C NMR (400 MHz, CHLOROFORM-*D*) δ 134.03, 129.73, 129.29, 127.85, 127.00, 126.26, 126.09, 125.88, 123.54, 29.90.

1,5-diacetylanthracene (**1,5-DAAN**) assignments. ^{1}H NMR (400 MHz, CHLOROFORM-*D*) δ 9.56 (s, 1H), 8.25 (dd, *J* = 8.4, 1.2 Hz, 1H), 8.08 (dd, *J* = 7.0, 1.2 Hz, 1H), 7.52 (dd, *J* = 8.5, 7.0 Hz, 1H), 2.81 (s, 3H). ^{13}C NMR (400 MHz, CHLOROFORM-*D*) δ 201.36, 135.29, 134.32, 133.49, 130.80, 127.83, 126.77, 124.25, 29.75.

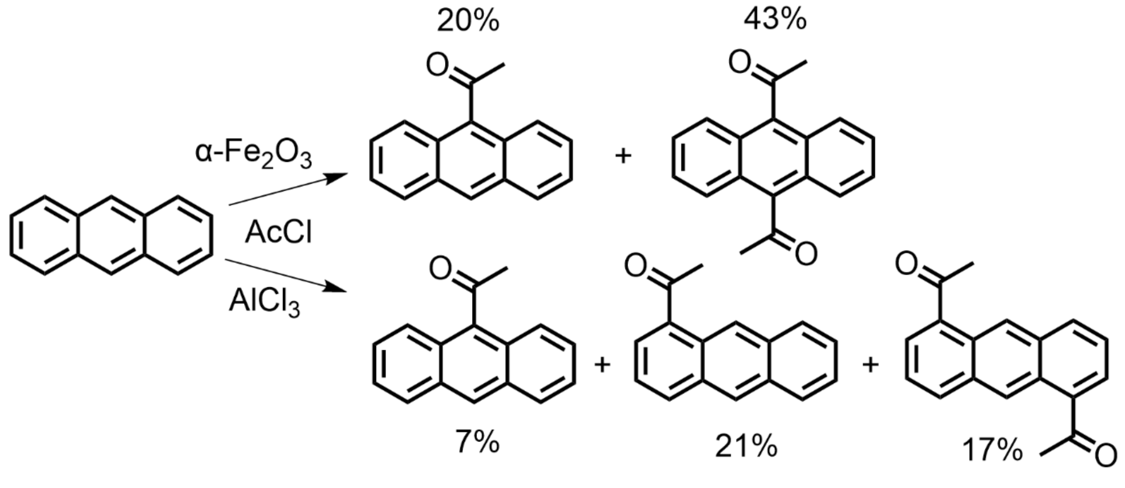

Scheme S1. Overview of synthesis approach for different acetylanthracenes with yields of each material shown.

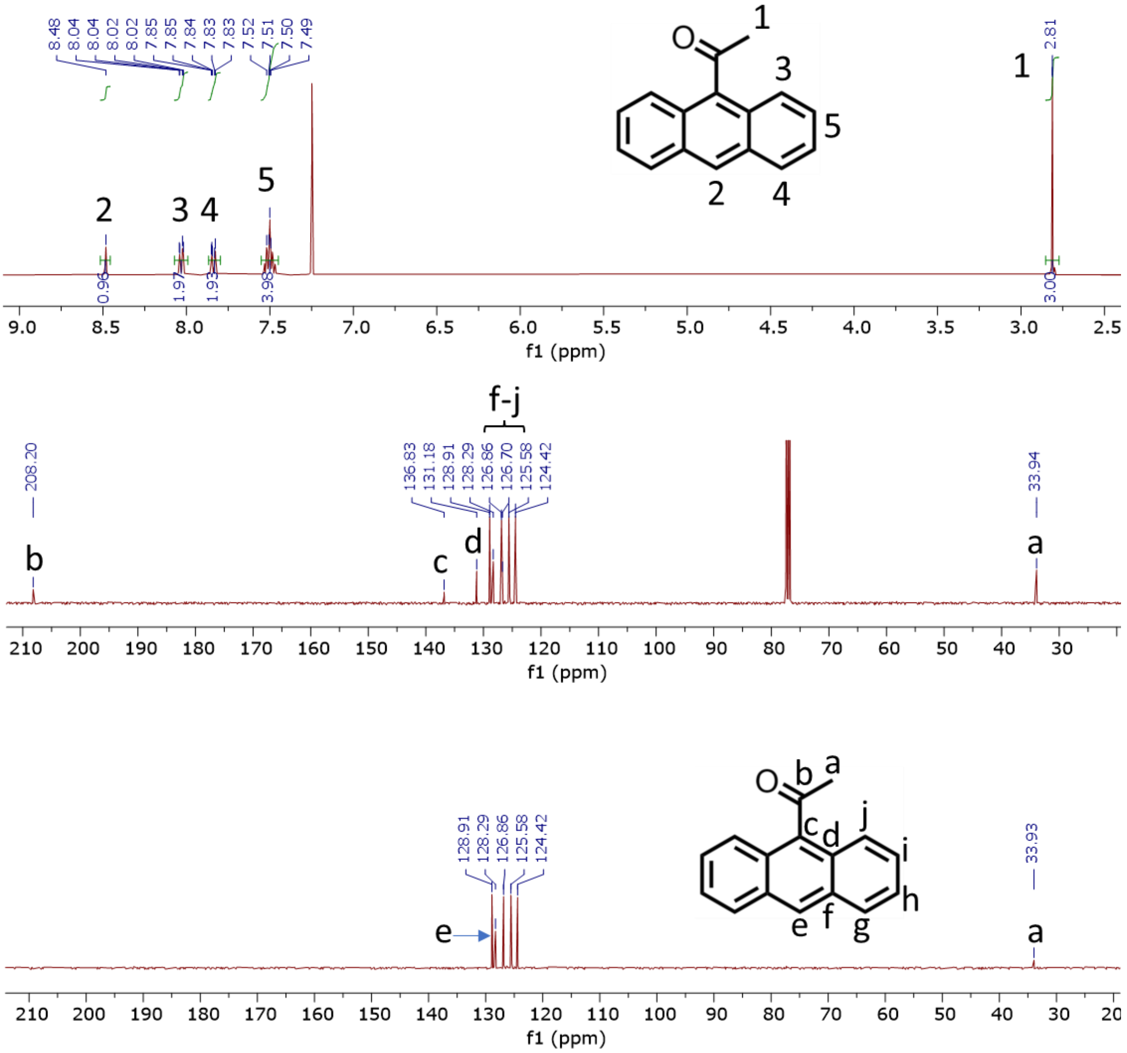

Figure S3. ^{1}H-NMR (top), ^{13}C (middle), DEPT (bottom) of 9-acetylanthracene (**9-AAN**).

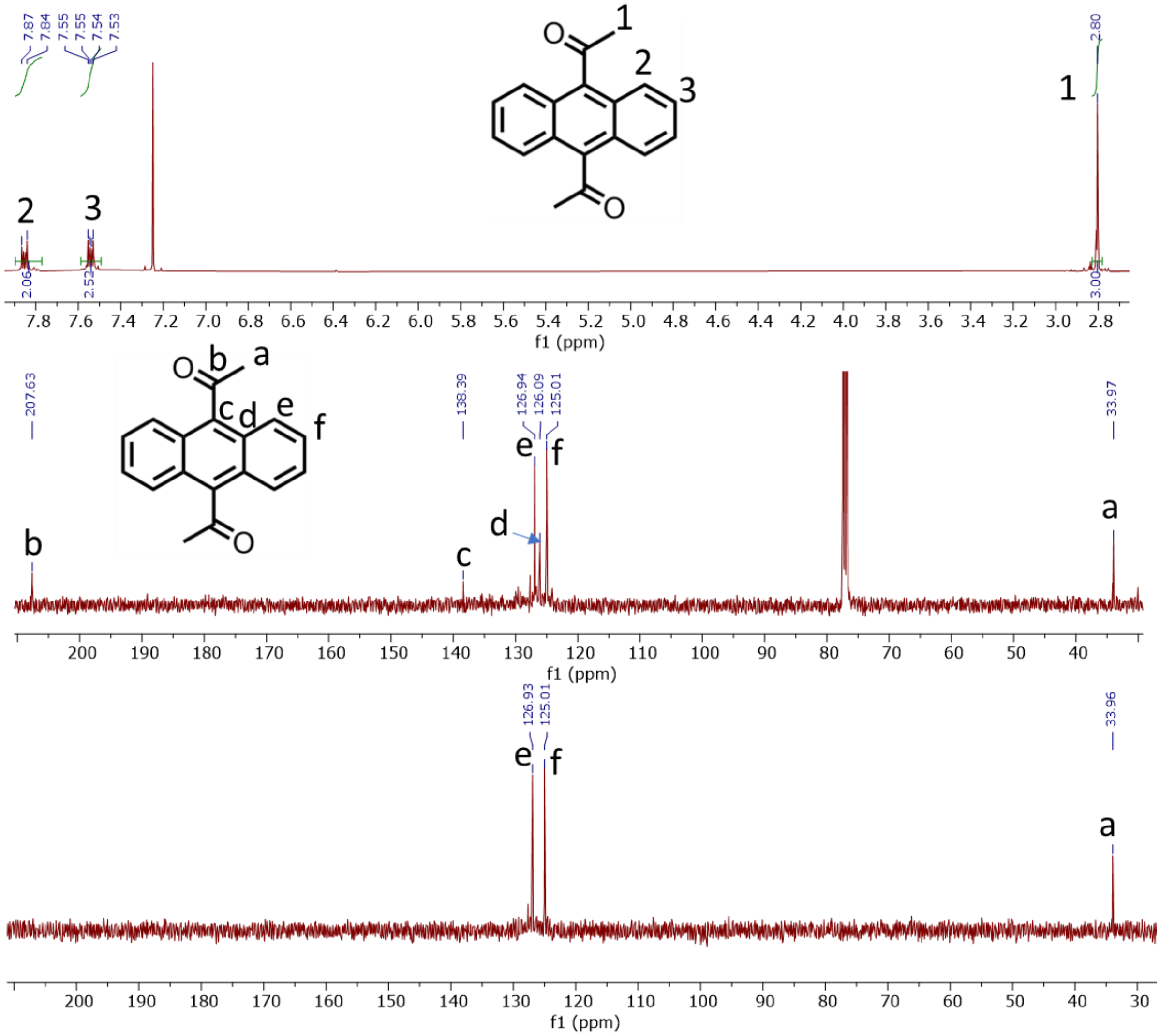

Figure S4. ^{1}H-NMR (top), ^{13}C (middle), DEPT (bottom) of 9,10-diacetylanthracene (**9,10-DAAN**).

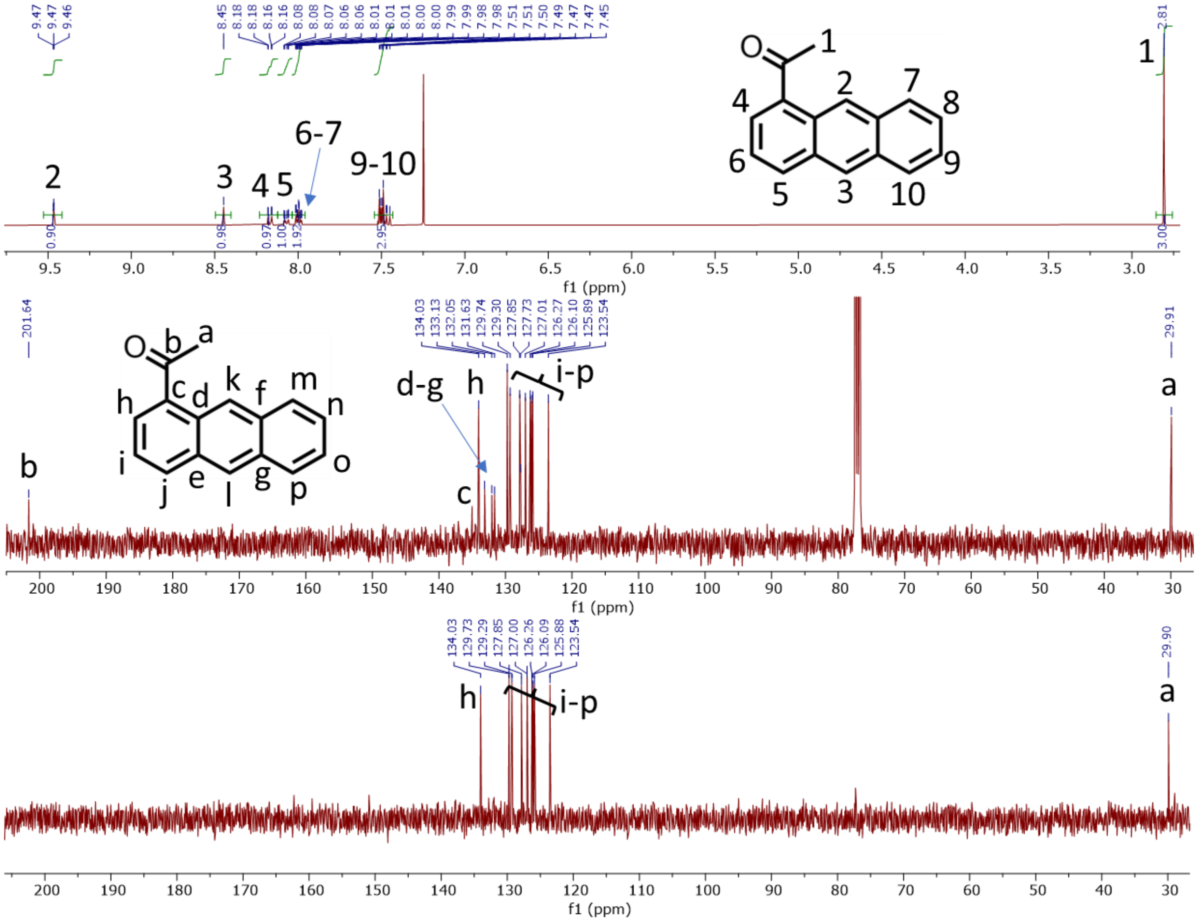

Figure S5. ^{1}H-NMR (top), ^{13}C (middle), DEPT (bottom) of 1-acetylanthracene (**1-AAN**).

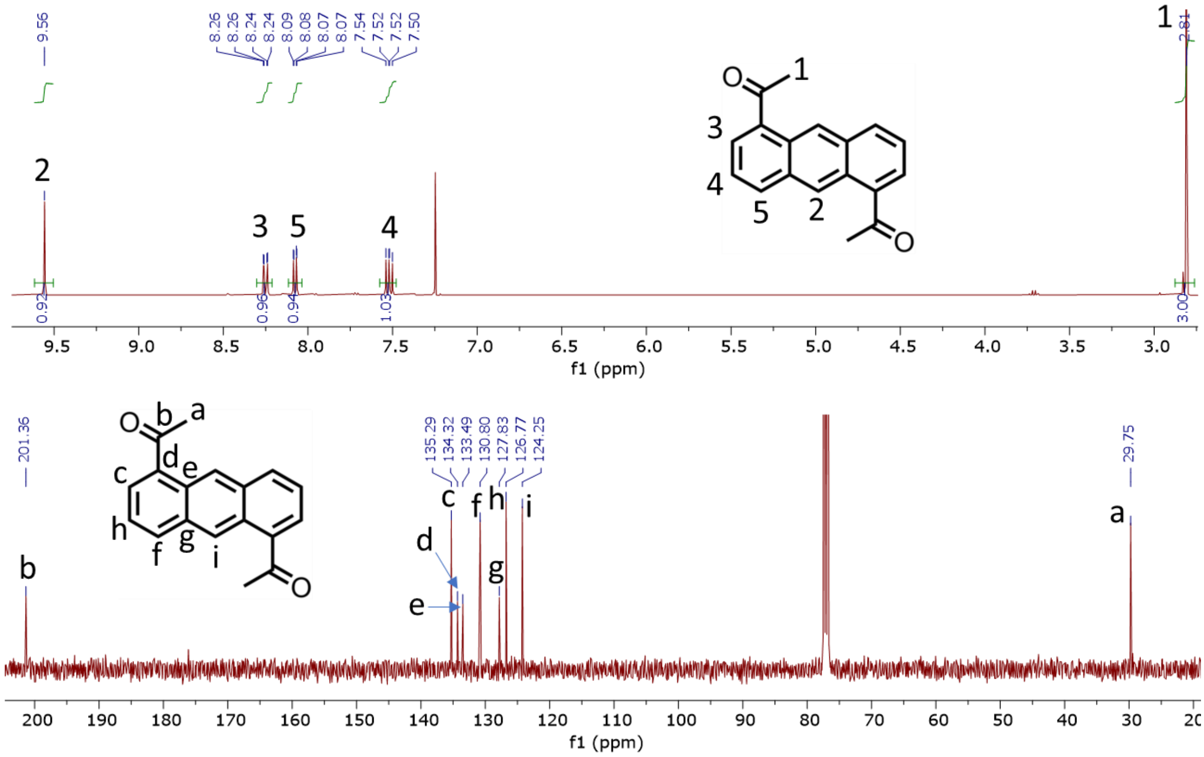

Figure S6. ^{1}H-NMR (top), ^{13}C (bottom) of 1,5-diacetylanthracene (1**,5-DAAN**).

EasySpin Fitting Details and Script

The least squares fitting of X-band triplet signals was performed using the "esfit" function inside the (V6.0.0 dev.51) package for MATLAB 2023a. The data was fitted with data "as is", and baseline "none", using the Nelder-Mead simplex algorithm. The following typical script used to load, and fit is given below (compatibility with EasySpin v6.0.0 dev.51):[4]

```
% Load in the Data
[B,spc, param] = eprload(['file_location.DTA']);

% Data Conversion – separation of real and imaginary data sets
spc_real = real(spc);
spc_imag = imag(spc);

% Background Correction - removal of laser noise by subtracting a time trace of a field position without sample-dependent signal
spc_real_corr = spc_real(:, :) - spc_real(:, 1);
Bmag = B{1,2};
Btime = B{1,1};

% Spin and experimental parameters
Sys = struct('S',1,'g',[2.001],'lw',[0.5 0.5], 'D',[1900 200]);
Sys.initState = {[0.1 0.8 0.1],'zerofield'};
Exp = struct('mwFreq',param.MWFQ*10^-9,'Harmonic',0);
Exp.Range = [273 423];

% Fitting Parameters
Vary = struct('lw',[2 2],'g',[0.01],'D',[350 100]);
Vary.initState = {[1 1 1], 'zerofield '};

% Least square fitting
esfit(spc_real_corr(50,:), @pepper, {Sys,Exp}, {Vary});
```

The signal maxima used for fitting were found by varying the time variable (#) and replotting the subsequent EPR trace:

```
figure(1)
plot(Bmag(:), Mspc(#,:));
hold on
```

The optimum time index can be checked by examining a field position (#) with a strong signal and looking for the highest signal, using the following script:

```
% Moving Average for Signal vs. Time @ Peak Positions if required
MmagLF = movmean(spc_real_corr(:,#), 5);    % Low-field signal
MmagHF = movmean(spc_real_corr(:,#), 5);    % High-field signal

% Plotting time vs Signal
figure(2)
plot(Btime*1000, MmagLF, 'r');
hold on
plot(Btime*1000, MmagHF, 'r');
```

During the fit, the minimum and maximum allowed values for "initState", which determines the triplet sublevel populations, were set to 0 and 1, respectively. The final simulated values for then normalised to a total of 1 to obtain the reported triplet populations. Similarly, the minimum value for "lw" was set to zero to prevent non-physical negative values from arising.

Pulsed EPR Data and Fitting

Pulsed EPR data was collected by measuring echoes with and without laser flashes (500 nm light, 3.5 – 4.5 mJ/pulse, 5-7 ns pulses). Echoes originating from the cavity could then be subtracted from the sample data following a laser pulse. We initiated our measurements with **Anthracene:TCNB** at 10 K, wherein field-swept echoes were used to find the field position with the most intense signal at 3000/3900 G (**Fig. S7**). Due to the nature of sub-level specific spin-lattice relaxation,[9] we opted to target the same triplet sub-level transition at these canonical $Y^{-/+}$ field positions for all of our materials. The low signal-to-noise ratio of these powder measurements prevented an extensive temperature-dependent exploration of their spin dynamics.

Transient echo traces were fitted with single exponential functions, and temperature-dependent trends in T_1 and T_2, were fitted to the appropriate Direct, Raman, or Orbach processes according to the following relationships:[10–12]

Direct Process, $$\frac{1}{T_1} = A_{dir}B^4T$$

Where T_1 is the spin-lattice relaxation, A_{dir} is an experimentally determined adjustable parameter, B is the magnetic field, and T is temperature.

Raman Process, $$\frac{1}{T_1} = b_oT^n$$

Where b_o is an experimentally determined adjustable parameter, T^n is the temperature with a power dependence, n.

Orbach Process, $$\frac{1}{T_1} = b_o\Delta_{orb}^3 e^{-\Delta_{orb}/k_BT}$$

Where Δ_{orb} is the energy of the excited state in question, k_B is the Boltzmann constant.

Anthracene:TCNB was fitted with a modified relationship to describe clearly diverging behavior:

$$T_{1\,total}^{-1} = T_{1\,direct}^{-1} + T_{1\,raman/orbach}^{-1}$$

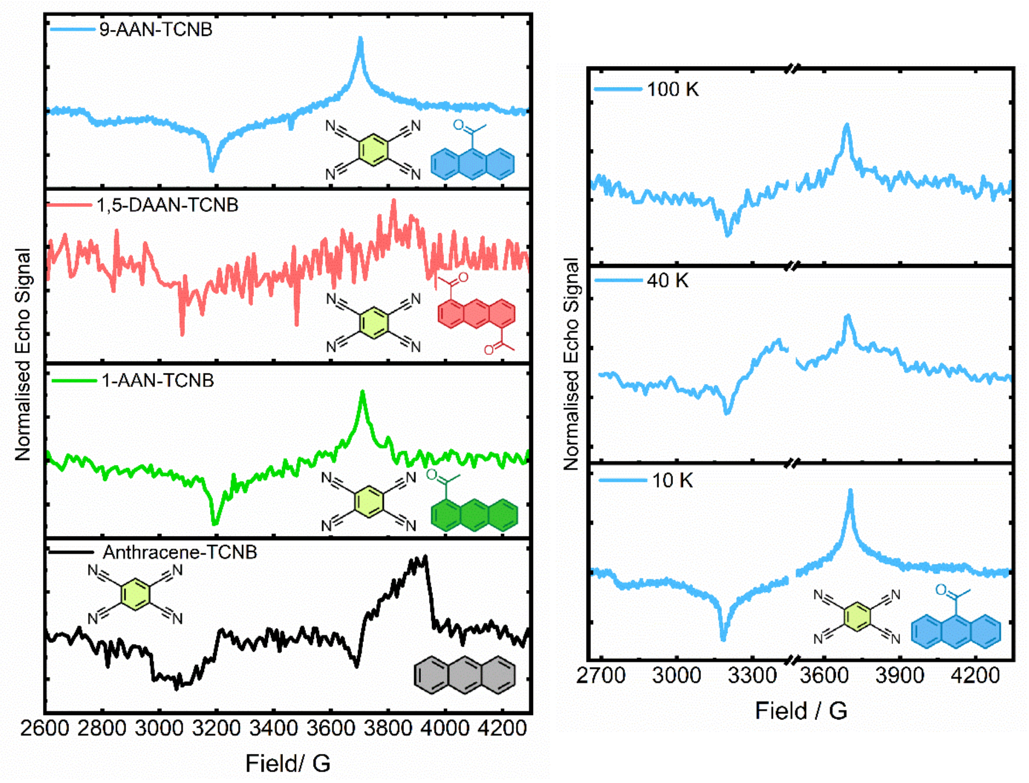

Figure S7. (left) Field swept echoes for each CT material measured at 10 K; (right) Field swept echoes of **9-AAN:TCNB** collected at different temperatures.

Single Crystal X-ray Diffraction

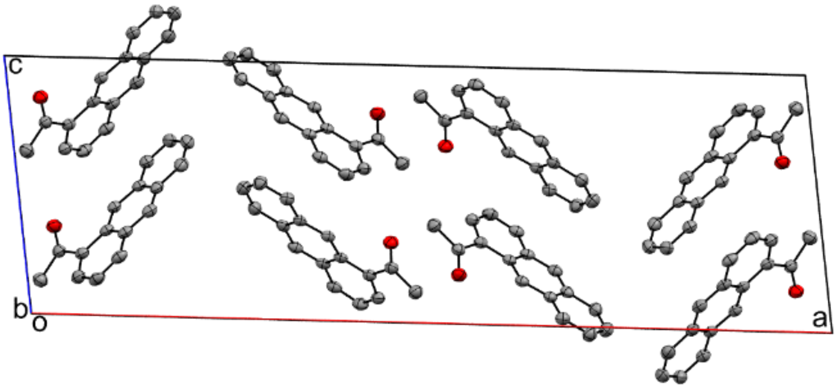

Figure S8: (**A**) Unit cell of **1-AAN** viewed along the b-axis. **1-AAN** crystallized in the *P2₁/c* space group.

Table S2. Crystal structure experimental and refinement parameters.

Name	Anthracene:TCNB	1-acetylanthracene	1-acetylanthracene:TCNB	1,5-diacetylanthracene:TCNB
CCSD numbers	2347419	2313864	2313865	2313866
Formula	$C_{10}H_2N_4$, $C_{14}H_{10}$	$C_{16}H_{12}O$	$C_{16}H_{12}O$, $C_{10}H_2N_4$	$C_{18}H_{14}O_2$, $C_{10}H_2N_4$
Formula weight	356.38	220.26	398.41	440.45
Temperature / K	173(2)	173(2)	173(2)	173(2) K
Diffractometer, λ / Å	0.71073	1.54184	1.54184	0.71073 Å
System, Space group	Monoclinic, $P2_1/c$	Monoclinic, $P2_1/c$	Triclinic, P-1	Triclinic, P-1
Unit cell Dimensions	a = 7.35613(15) Å, b = 12.7065(3) Å c = 9.48252(16) Å	a = 34.1490(15) Å b = 6.0718(2) Å c = 11.0104(4) Å	a = 6.7716(5) Å b = 7.9726(9) Å c = 19.1203(14) Å	a = 7.2411(4) Å b = 7.9272(3) Å c = 9.8383(5) Å
	α = 90°, β = 92.8209(17)° λ = 90°	α = 90°, β = 97.393(4)° λ = 90°	α = 91.247(7)°, β = 98.806(6)°, λ = 102.657(7)°	α = 88.598(4)°, β = 86.018(4)°, λ = 77.299(4)°
Volume, Z / Å^3	885.26(3), 2	2263.98(16), 8	993.66(15), 2	549.56(5), 1
Density (calculated) / gcm^{-3}	1.337	1.292	1.332	1.331
Absorption coeff. / mm^{-1}	0.082	0.618	0.673	0.087
F(000)	368	928	412	228
Crystal colour / morphology	Orange blocks	Pale yellow plates	Yellow platy needles	Yellow blocky needles
Crystal size / mm^3	0.604 x 0.422 x 0.323	0.349 x 0.257 x 0.023	0.290 x 0.106 x 0.025	0.523 x 0.408 x 0.329
q range for data collection	2.682 to 28.275°	3.916 to 73.539°	4.688 to 73.880°	2.634 to 28.333°
Index ranges	-9<=h<=9, -16<=k<=16, -12<=l<=12	-40<=h<=41, -7<=k<=7, -9<=l<=13	-8<=h<=5, -9<=k<=9, -23<=l<=23	-9<=h<=9, -10<=k<=9, -12<=l<=12
Reflns collected / unique	46384 / 2083 [R(int) = 0.0408]	13409 / 4467 [R(int) = 0.0443]	7373 / 7373 [R(int) = 0.0302]	6885 / 2314 [R(int) = 0.0186]
Reflns observed [F>4s(F)]	1645	2939	4065	1947
Absorption correction	Analytical	Analytical	Analytical	Analytical
Max. and min. transmission	0.979 and 0.966	0.986 and 0.866	0.984 and 0.903	0.979 and 0.968
Goodness-of-fit on F2	1.059	1.029	0.967	1.043
Final R indices [F>4s(F)]	R1 = 0.0476, wR2 = 0.1278	R1 = 0.0448, wR2 = 0.1049	R1 = 0.0502, wR2 = 0.1411	R1 = 0.0405, wR2 = 0.1048
R indices (all data)	R1 = 0.0611, wR2 = 0.1394	R1 = 0.0776, wR2 = 0.1255	R1 = 0.0976, wR2 = 0.1676	R1 = 0.0490, wR2 = 0.1110

Additional DFT Calculations

Computational electronic calculations were performed on acetylanthracenes and their co-crystal dimers at the CAM-B3LYP/6-311G+d level[13] in Gaussian09.[14,15] The molecules were initially optimised for the single ground state. The ground state geometries of co-crystal dimer structures were taken from their respective crystal structures. **9-AAN:TCNB** was taken from the structure reported by Li *et al.*[16] To determine the singlet and triplet state energies of the dimers, time-dependent-density functional theory (TD-DFT) calculations were performed on the singlet state geometry. All excitation energies were normalized to the singlet ground state following Bogatko *et al.*[17]

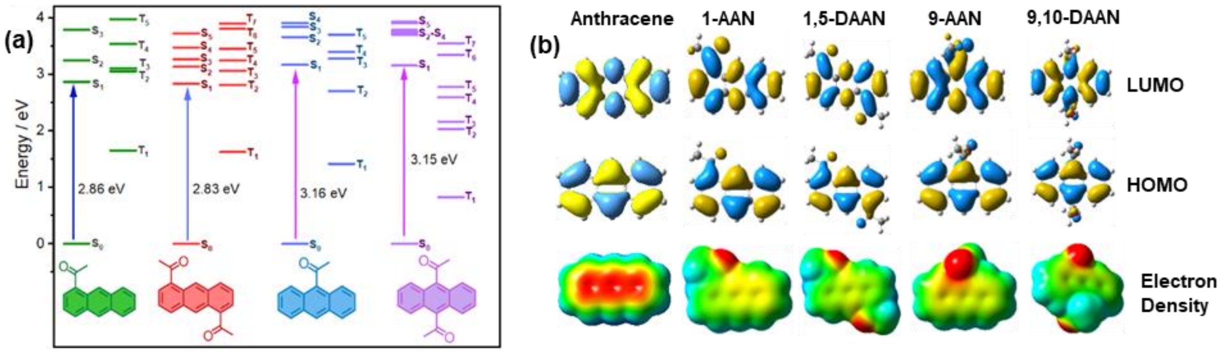

Figure S9: (**a**) Singlet and triplet state energies of optimized acetylanthracenes; (**b**) their HOMO (S_0) and LUMO (S_1) orbitals and ground state total electron density distributions.

Table S3: Summary of DFT-derived molecular state energies. Energies are normalized against the energy of S_0. Units in eV.

**Compound**	**Exp. S_0 – S_1**	**S_1**	**T_n**	**T_1**	**$\Delta E_{S1\text{-}Tn}$**	**$\Delta E_{S1\text{-}T1}$**
**1-AAN**	2.86	3.21	3.47	2.08	0.25	1.21
**1,5-DAAN**	2.83	3.14	3.28	2.09	0.14	1.05
**9-AAN**	3.16	3.39	3.75	2.11	0.36	1.28
**9,10-DAAN**	3.15	3.37	3.77	2.24	0.40	1.13
**Anthracene:TCNB**	2.42	2.64	2.51	2.33	0.13	0.31
**1-AAN:TCNB**	2.48	2.57	2.7	2.18	0.13	0.39
**1,5-DAAN:TCNB**	2.61	2.78	2.77	2.27	0.01	0.51
**9-AAN:TCNB**	2.48	2.78	2.81	2.28	-0.03	0.50

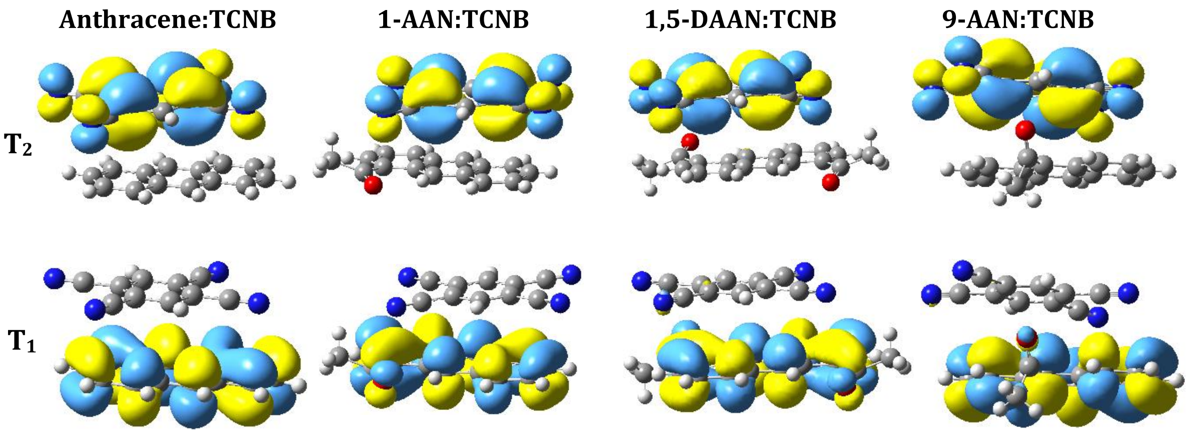

Figure S10: Molecular orbitals for low-lying triplet states for (**a**) **Anthracene:TCNB**, (**b**) **1-AAN:TCNB**, (**c**) **1,5-DAAN:TCNB**, (**d**) **9-AAN:TCNB**

Spin-orbit Coupling Calculations

The spin-orbit coupling matrix elements were determined at the B3LYP def2-SVP level using the Orca 5.0.1 package.[18,19]

Table S4: Spin-orbit coupling matrix elements for **Anthracene** and its **TCNB** co-crystal determined by TD-DFT at the B3LYP/def2-ZVP level.

**Triplet State, n**	**Singlet State, n**	**Anthracene** $\langle T\|H_{SO}\|S\rangle$ / cm^{-1}			**Anthracene:TCNB** $\langle T\|H_{SO}\|S\rangle$ / cm^{-1}		
		**X**	**X**	**Z**	**X**	**X**	**Z**
1	0	0	0	0	0	0.3	0
1	1	0	0	0	0	0	0
1	2	0	0	-0.04	-0.04	0	0.08
1	3	0	-0.01	0	-0.02	0	-0.04
1	4	-0.01	0	0	-0.5	0	0.16
1	5	0	0	0	0	0.14	0
2	0	-0.01	0	0.02	0.17	0	-0.08
2	1	-0.01	0	0	-0.28	0	0.1
2	2	0	0	0	0	-0.12	0
2	3	0	0	0	0	0.03	0
2	4	0	0	0	0	0.06	0
2	5	0	-0.01	0	-0.05	0	0.02
3	0	0	-0.01	0	-0.1	0	0.05
3	1	0	0	0.05	-0.09	0	-0.03
3	2	0	0	0	0	-0.03	0
3	3	0	0	0	0	0	0
3	4	0	0	0	0	-0.09	0
3	5	0	0	0.01	-0.01	0	0
4	0	-0.01	0	0	0.04	0	-0.01
4	1	0	0	0.05	0.07	0	-0.03
4	2	0	0	0	0	0.12	0
4	3	0	0	0	0	-0.01	0
4	4	0	0	0	0	0.04	0
4	5	0	0	-0.01	0.08	0	-0.02
5	0	0	0	0	-0.02	0	0.02
5	1	0	0	0	0.04	0	-0.05
5	2	0	0	0.01	0	-0.02	0
5	3	0	0	0	0	0.01	0
5	4	0.01	-0.01	0	0	-0.05	0
5	5	0	0	0	0.1	0	-0.03

Table S5: Spin-orbit coupling matrix elements for 1-acetylanthracene (**1-AAN**) and its **TCNB** co-crystal determined by TD-DFT at the B3LYP/def2-ZVP level.

Triplet State, n	Singlet State, n	1-AAN <T|H$_{SO}$|S> / cm^{-1}			1-AAN:TCNB <T|H$_{SO}$|S> / cm^{-1}		
		X	X	Z	X	X	Z
1	0	0	0	0.01	-0.01	-0.18	-0.01
1	1	0	0	0	-0.01	0.19	-0.11
1	2	10.11	1.17	0	-0.01	0.01	0.14
1	3	0	0	-0.03	0.18	-0.26	-0.41
1	4	0	0	0.01	0.67	-0.98	-1.6
1	5	0	0	0.02	0.58	-0.83	-1.46
2	0	45.74	5.92	0.01	0.01	-0.22	-0.26
2	1	4.99	0.51	0	0.01	0.02	-0.02
2	2	0	0	0.37	0.03	-0.16	-0.23
2	3	-2.05	-0.45	0	-2.56	3.93	6.08
2	4	-0.09	0.03	0	0.16	-0.43	-0.23
2	5	-10.6	-1.68	0	-0.01	0.09	0.15
3	0	0	0	0	0.01	0.09	0.15
3	1	0	0	-0.01	-0.03	-0.1	-0.08
3	2	-13.97	-1.74	-0.01	0	-0.01	-0.03
3	3	0	0	-0.01	0	0.02	0.01
3	4	0	0	0	0.04	-0.12	-0.03
3	5	0	0	0	0	0.02	0.01
4	0	0	0	0	-0.91	1.8	2.9
4	1	0	0	0.02	2.66	-4.08	-6.29
4	2	1.38	0.28	0	0.32	-0.48	-0.75
4	3	0	0	0	0	0.01	-0.02
4	4	0	0	0	0.5	-0.95	-1.35
4	5	0	0	0	-0.49	1.18	1.47
5	0	0	0	-0.04	-10.18	20.38	33
5	1	0	0	-0.04	-0.14	0.19	0.38
5	2	8.36	1.16	0	-0.27	0.39	0.63
5	3	0	0	-0.01	-0.08	0.21	-0.04
5	4	0	0	-0.03	1.81	-2.54	-3.87
5	5	0	0	0	1.33	-1.87	-2.86

Table S6: Spin-orbit coupling matrix elements for 1,5-diacetylanthracene (**1,5-DAAN**) and its **TCNB** co-crystal determined by TD-DFT at the B3LYP/def2-ZVP level.

**Triplet State, n**	**Singlet State, n**	**1,5-DAAN** <T|H_{SO}|S> / cm^{-1}			**1,5-DAAN:TCNB** <T|H_{SO}|S> / cm^{-1}		
		**X**	**X**	**Z**	**X**	**X**	**Z**
1	0	0	0	0	0.03	-0.26	-0.4
1	1	0	0	0	-0.1	0.1	-0.14
1	2	-13.37	-1.46	-0.01	-0.02	0.06	-0.29
1	3	0	0	0	0.63	-0.88	-1.24
1	4	0	0	0	0.93	-1.15	-1.87
1	5	0	0	0.03	-0.08	0.1	0.17
2	0	0.02	0	0	-0.13	0.44	-0.09
2	1	0	0	0	0.04	-0.05	-0.01
2	2	0	0	0	-0.01	0.2	0.24
2	3	17.49	2	0.01	3.45	-4.72	-6.54
2	4	0	0	0	0.14	-0.22	0.11
2	5	0	0	0	1.56	-2.07	-3.01
3	0	0.01	0	0.01	-0.12	0.31	0.64
3	1	7.8	0.84	0	-0.11	0.33	0.2
3	2	0	0	0.37	-0.17	0.31	0.19
3	3	-0.01	0	0	0	0.05	0.02
3	4	0	0	0	-0.4	-0.65	-0.42
3	5	-2.22	-0.51	0	0.08	-0.17	-0.16
4	0	64.86	7.79	0.03	-2.22	4.55	6.53
4	1	0	0	0	-3.43	4.71	6.5
4	2	0	0	0	-0.01	0.01	-0.01
4	3	0	0	0.34	0.01	-0.01	-0.02
4	4	-2.86	-0.24	0	-0.51	0.65	1
4	5	0	0	0	0.12	-0.22	-0.25
5	0	0	0	0	-3.36	8.32	12.38
5	1	0	0	0.02	0.64	-0.97	-1.49
5	2	-1.71	-0.09	0	-0.73	1.01	1.21
5	3	0	0	0	-0.24	0.32	0.42
5	4	0	0	0	-1.02	1.33	1.99
5	5	0	0	0	0.33	-0.4	-0.64

Table S7: Spin-orbit coupling matrix elements for 9-acetylanthracene (**9-AAN**) and its **TCNB** co-crystal determined by TD-DFT at the B3LYP/def2-ZVP level.

**Triplet State, n**	**Singlet State, n**	**9-AAN** <T|H_{SO}|S> / cm^{-1}			**9-AAN:TCNB** <T|H_{SO}|S> / cm^{-1}		
		**X**	**X**	**Z**	**X**	**X**	**Z**
1	0	0.09	-0.99	-1.67	0.14	-0.07	-0.56
1	1	0.44	0.32	-0.01	-0.3	-0.06	0.17
1	2	3.06	2.55	5.3	0.16	0.07	-0.22
1	3	0.16	0.12	1.19	0.32	-0.03	-0.16
1	4	-1.16	-0.5	2.79	0.3	-0.02	-0.29
1	5	-1.77	-0.79	-1.78	0.79	-0.03	-1.03
2	0	-0.02	33.73	35	0.25	0.27	0.07
2	1	2.85	1.49	3.29	0.11	0.02	-0.07
2	2	0.27	-1.36	-2.78	-0.09	0.02	0.08
2	3	-0.89	-0.41	-1.31	0.44	0.06	-0.24
2	4	-2.06	-0.86	-4.41	0.57	0.02	-0.75
2	5	-3.1	-1.39	0.21	2.53	-0.1	-3.29
3	0	0.31	-7.21	-7.23	-0.44	0.03	0.49
3	1	-0.62	-0.18	-0.24	-0.02	-0.06	0.05
3	2	-0.47	0.41	0.9	0.12	-0.01	-0.13
3	3	0.19	0.04	0.35	-0.25	0.02	0.29
3	4	0.71	-0.01	1.05	-0.2	-0.01	0.23
3	5	0.88	0.31	0.07	0.12	0.02	-0.14
4	0	0.71	-2.77	-2.97	-0.11	0.02	0.15
4	1	-0.17	-0.12	0.23	-0.24	0.03	0.23
4	2	-0.18	0.33	1.08	-0.21	0.01	0.21
4	3	0.08	0.1	0.22	-0.05	-0.11	0.07
4	4	-0.18	0.33	0.53	0.03	0.06	0
4	5	0.49	0.68	0.93	-0.02	-0.17	0.04
5	0	2.54	8.81	5.14	-0.21	0.61	0.17
5	1	-0.37	0.11	2.44	-0.04	-0.03	-0.22
5	2	2.63	3.98	5.99	0.03	0	-0.01
5	3	0.48	0.08	0.6	-0.1	0.03	0.01
5	4	-1.5	-0.4	-1.44	-0.09	0	-0.02
5	5	1.31	3.06	5.7	-0.51	0.05	-0.25

Table S8: Spin-orbit coupling matrix elements for 9,10-diacetylanthracene (**9,10-DAAN**) determined by TD-DFT at the B3LYP/def2-ZVP level.

Triplet state, n	Singlet State, n	<T|H_{SO}|S> / cm^{-1}		
		X	Y	Z
1	0	0.52	-0.97	2.44
1	1	0.49	0.28	0.01
1	2	4.02	2.34	0.07
1	3	-1.17	2.04	-1.09
1	4	0.51	0.3	0.01
1	5	-1.18	2.09	-2.58
2	0	-14.84	26.54	-42.51
2	1	2.18	1.28	0.04
2	2	-0.01	0	0
2	3	0.04	-0.07	0.47
2	4	-1.57	-0.92	-0.02
2	5	0.34	-0.62	1.26
3	0	13.13	-23.29	36.8
3	1	-1.74	-1.03	-0.02
3	2	-0.82	-0.48	-0.03
3	3	-0.02	0.03	-0.94
3	4	2.04	1.2	0
3	5	-0.23	0.44	-1.92
4	0	-13.36	-8.05	0.08
4	1	-1.05	1.78	-1.06
4	2	0.02	-0.14	3.43
4	3	1.85	1.09	0.02
4	4	0.52	-0.97	4.26
4	5	0.19	0.12	-0.02
5	0	1.75	1.03	0.01
5	1	0.06	-0.08	-0.58
5	2	0.07	-0.1	-0.86
5	3	0.04	0.02	0
5	4	-0.02	0.05	-0.42
5	5	0.08	0.05	0

References

(1) Dolomanov, O. V.; Bourhis, L. J.; Gildea, R. J.; Howard, J. A. K.; Puschmann, H. OLEX2 : A Complete Structure Solution, Refinement and Analysis Program. *J Appl Crystallogr* **2009**, *42* (2), 339–341. https://doi.org/10.1107/S0021889808042726.

(2) *SHELXTL v5.1*; Madison, WI, 53711, 1997. https://doi.org/10.1049/esn.1987.0025.

(3) Sheldrick, G. M. Crystal Structure Refinement with SHELXL. *Acta Crystallogr C Struct Chem* **2015**, *71* (1), 3–8. https://doi.org/10.1107/S2053229614024218.

(4) Stoll, S.; Schweiger, A. EasySpin, a Comprehensive Software Package for Spectral Simulation and Analysis in EPR. *Journal of Magnetic Resonance* **2006**, *178* (1), 42–55. https://doi.org/10.1016/j.jmr.2005.08.013.

(5) Šimėnas, M.; O'Sullivan, J.; Zollitsch, C. W.; Kennedy, O.; Seif-Eddine, M.; Ritsch, I.; Hülsmann, M.; Qi, M.; Godt, A.; Roessler, M. M.; Jeschke, G.; Morton, J. J. L. A Sensitivity Leap for X-Band EPR Using a

Probehead with a Cryogenic Preamplifier. *Journal of Magnetic Resonance* **2021**, *322*, 106876. https://doi.org/10.1016/j.jmr.2020.106876.

(6) Schröder, M.; Rauber, D.; Matt, C.; Kay, C. W. M. Pentacene in 1,3,5-Tri(1-Naphtyl)Benzene: A Novel Standard for Transient EPR Spectroscopy at Room Temperature. *Appl Magn Reson* **2022**, *53* (7–9), 1043–1052. https://doi.org/10.1007/s00723-021-01420-4.

(7) Bassilios, H. F.; Shawky, M.; Salem, A. Y. Acetylation of Anthracene by the Friedel-crafts Reaction Using Chloroform as the Solvent. *Recueil des Travaux Chimiques des Pays-Bas* **1962**, *81* (8), 679–682. https://doi.org/10.1002/recl.19620810806.

(8) Philip, A. M.; Gudem, M.; Sebastian, E.; Hariharan, M. Decoding the Curious Tale of Atypical Intersystem Crossing Dynamics in Regioisomeric Acetylanthracenes. *Journal of Physical Chemistry A* **2019**, *123* (29), 6105–6112. https://doi.org/10.1021/acs.jpca.9b00766.

(9) Wu, H.; Ng, W.; Mirkhanov, S.; Amirzhan, A.; Nitnara, S.; Oxborrow, M. Unraveling the Room-Temperature Spin Dynamics of Photoexcited Pentacene in Its Lowest Triplet State at Zero Field. *Journal of Physical Chemistry C* **2019**, *123* (39), 24275–24279. https://doi.org/10.1021/acs.jpcc.9b08439.

(10) Mitrikas, G.; Sanakis, Y.; Raptopoulou, C. P.; Kordas, G.; Papavassiliou, G. Electron Spin–Lattice and Spin–Spin Relaxation Study of a Trinuclear Iron(<scp>iii</Scp>) Complex and Its Relevance in Quantum Computing. *Phys. Chem. Chem. Phys.* **2008**, *10* (5), 743–748. https://doi.org/10.1039/B711056A.

(11) Eaton, S. S.; Eaton, G. R. Relaxation Times of Organic Radicals and Transition Metal Ions. In *Biomedical EPR, Part B: Methodology, Instrumentation, and Dynamics*; 2002; Vol. 19, pp 29–154. https://doi.org/10.1007/0-306-47109-4_2.

(12) Harbridge, J. R.; Eaton, S. S.; Eaton, G. R. Electron Spin-Lattice Relaxation Processes of Radicals in Irradiated Crystalline Organic Compounds. *J Phys Chem A* **2003**, *107* (5), 598–610. https://doi.org/10.1021/jp021504h.

(13) Yanai, T.; Tew, D. P.; Handy, N. C. A New Hybrid Exchange–Correlation Functional Using the Coulomb-Attenuating Method (CAM-B3LYP). *Chem Phys Lett* **2004**, *393* (1–3), 51–57. https://doi.org/10.1016/j.cplett.2004.06.011.

(14) Frisch, M. J.; Trucks, G. W.; Schlegel, H. B.; Scuseria, G. E.; Robb, M. A.; Cheeseman, J. R.; Scalmani, G.; Barone, V.; Mennucci, B.; Petersson, G. A.; Nakatsuji, H.; Caricato, M.; Li, X.; Hratchian, H. P.; Izmaylov, A. F.; J. Bloino, G. Z.; Sonnenberg, J. L.; Hada, M.; Ehara, M.; Toyota, K.; Fukuda, R.; Hasegawa, J.; Ishida, M.; Nakajima, T.; Honda, Y.; Kitao, O.; Nakai, H.; Vreven, T.; J. A. Montgomery, Jr.; Peralta, J. E.; Ogliaro, F.; Bearpark, M.; Heyd, J. J.; Brothers, E.; Kudin, K. N.; Staroverov, V. N.; Kobayashi, R.; Normand, J.; Raghavachari, K.; Rendell, A.; Burant, C. J.; Iyengar, S. S.; Tomasi, J.; Cossi, M.; Rega, N.; Millam, J. M.; Klene, M.; Knox, J. E.; Cross, J. B.; Bakken, V.; Adamo, C.; Jaramillo, J.; Gomperts, R.; Stratmann, R. E.; Yazyev, O.; Austin, A. J.; Cammi, R.; Pomelli, C.; Ochterski, J. W.; Martin, R. L.; Morokuma, K.; Zakrzewski, V. G.; Voth, G. A.; Salvador, P.; Dannenberg, J. J.; Dapprich, S.; Daniels, A. D.; Farkas, Ö.; Foresman, J. B.; Ortiz, J. V.; J. Cioslowski; Fox, D. J. *Gaussian 09*; Gaussian, Inc.: Wallingford CT, 2009.

(15) Daga, L. E.; Civalleri, B.; Maschio, L. Gaussian Basis Sets for Crystalline Solids: All-Purpose Basis Set Libraries vs System-Specific Optimizations. *J Chem Theory Comput* **2020**, *16* (4), 2192–2201. https://doi.org/10.1021/acs.jctc.9b01004.

(16) Li, S.; Lin, Y.; Yan, D. Two-Component Molecular Cocrystals of 9-Acetylanthracene with Highly Tunable One-/Two-Photon Fluorescence and Aggregation Induced Emission. *J Mater Chem C Mater* **2016**, *4* (13), 2527–2534. https://doi.org/10.1039/C6TC00067C.

(17) Bogatko, S.; Haynes, P. D.; Sathian, J.; Wade, J.; Kim, J. S.; Tan, K. J.; Breeze, J.; Salvadori, E.; Horsfield, A.; Oxborrow, M. Molecular Design of a Room-Temperature Maser. *Journal of Physical Chemistry C* **2016**, *120* (15), 8251–8260. https://doi.org/10.1021/acs.jpcc.6b00150.

(18) Neese, F. The ORCA Program System. *Wiley Interdiscip Rev Comput Mol Sci* **2012**, *2* (1), 73–78. https://doi.org/10.1002/wcms.81.

(19) Neese, F. Calculation of the Zero-Field Splitting Tensor on the Basis of Hybrid Density Functional and Hartree-Fock Theory. *Journal of Chemical Physics* **2007**, *127* (16). https://doi.org/10.1063/1.2772857.
